# A Versatile ΦC31 Based Reporter System for Measuring AP-1 and Nrf2 Signaling in *Drosophila* and in Tissue Culture

**DOI:** 10.1371/journal.pone.0034063

**Published:** 2012-04-11

**Authors:** Nirmalya Chatterjee, Dirk Bohmann

**Affiliations:** Department of Biomedical Genetics, University of Rochester Medical Center, Rochester, New York, United States of America; University College London, United Kingdom

## Abstract

This paper describes the construction and characterization of a system of transcriptional reporter genes for monitoring the activity of signaling pathways and gene regulation mechanisms in intact *Drosophila*, dissected tissues or cultured cells. Transgenic integration of the reporters into the *Drosophila* germline was performed in a site-directed manner, using ΦC31 integrase. This strategy avoids variable position effects and assures low base level activity and high signal responsiveness. Defined integration sites furthermore enable the experimenter to compare the activity of different reporters in one organism. The reporter constructs have a modular design to facilitate the combination of promoter elements (synthetic transcription factor binding sites or natural regulatory sequences), reporter genes (eGFP, or DsRed.T4), and genomic integration sites. The system was used to analyze and compare the activity and signal response profiles of two stress inducible transcription factors, AP-1 and Nrf2. To complement the transgenic reporter fly lines, tissue culture assays were developed in which the same synthetic ARE and TRE elements control the expression of firefly luciferase.

## Introduction

Signal transduction mechanisms that modulate transcription profiles in response to extracellular inputs control all major biological processes, including development, growth and homeostasis. Our knowledge of signal-dependent transcription control has been derived from studies *in vitro* and in cultured cells, and from genetic experiments in model organisms. Such research has yielded detailed molecular descriptions of many signaling and gene regulation processes. Questions that are, however, much less well understood include: How are spatial and temporal patterns of gene expression regulated in the two and three dimensions of epithelia or tissues? How do multiple signaling systems and transcription factors interact and form complex gene control networks in the intact organism? Addressing such questions requires experimental tools with which the activity of signal-dependent transcription factors can be monitored in live tissues with good temporal and spatial resolution. To this end, reporter genes that can register the activity of transcription factors or other signaling proteins have been designed. A widespread type of reporter relies on fusion genes in which synthetic DNA elements, that represent defined binding sites for a transcription factor of interest, are inserted upstream of sequences encoding protein products such as fluorescent proteins and enzymes, which are easy to visualize or quantify, are not normally present in the test organism, and for which sensitive assays are available such as chloramphenicol acetyl transferase or luciferase. Such reporter genes have been used in tissue culture or in transgenic animals [1]. Transcriptional *Drosophila* GFP or RFP reporters have been employed in the analysis of specific signaling pathways and regulatory processes in the context of an intact organism [2].

One technical limitation of most transgenic *Drosophila* reporter lines published so far is that they have been generated by random P-element integration into the genome of a recipient strain. Consequently, the insertion sites and their genomic environment are undefined and these reporters can be subject to variable and unpredictable position effects. The influence of nearby gene regulatory elements or the overall epigenetic state at the integration site can substantially affect the expression characteristics and response patterns of transgenes [3], [4]. Such inherent variability due to different insertion sites complicates comparisons between reporters. It is therefore difficult to directly relate the activity and responsiveness of a set of reporter mutants or to generate negative control reporters that differ from experimental reporters only by disruption of a DNA binding element.

The development of targeted methods for site specific transgene integration into the *Drosophila* germline can alleviate problems arising from random position effects. The feasibility of such strategies has recently been documented [5], [6]. We therefore used a targeted reporter gene insertion strategy that relies on the ΦC31 method [7]. The modular system of reporters uses eGFP, or DsRed.T4 constructs that are integrated in defined genomic locations. Corresponding firefly luciferase reporter constructs for cell culture experiments were generated as well, and can complement *in vivo* assays by providing platforms for rapid quantitative analyses and high throughput screens. This standardized system permits comparative reporter analysis across different signaling pathways using *in vivo* and *in vitro* platforms.

## Results

The continued integrity of cells and organisms depends on their ability to protect themselves against oxidative stress and fluctuations in redox state. Multiple signaling systems have evolved that can sense oxidative and genotoxic stresses and orchestrate rapid and effective responses in order to deflect or repair potentially harmful damage to cells, tissues and organisms. Conserved stress-inducible signaling systems include: the JNK, p38, p53, NF-kB and Nrf2 pathways (reviewed in [8]). The molecular constituents that relay stress signals and their epistatic relationship are, in most cases, well described. However, the interplay among these pathways, signal response profiles and biological functions in the organism are not comprehensively understood.

To begin a systematic comparative analysis of stress signaling pathways in specific organ systems and developmental processes, we generated a modular reporter system for monitoring the activities of two major stress responsive signaling pathways, the JNK and Nrf2 cascades, in *Drosophila*.

JNK (c-Jun NH_2_-terminal Kinase) is the eponymous enzyme of a MAP kinase pathway that has been implicated in stress responses, embryonic development, wound healing, apoptosis and cancer [9], [10], [11], [12]. JNK activity can be induced by multiple signals, including oxidative stress, UV radiation, inflammatory cytokines and epithelial wounding. The prototype substrate for JNK is the AP-1 transcription factor c-Jun, which upon binding to JNK and phosphorylation at several N-terminally located amino acid residues acquires the ability to activate its target genes as a heterodimer with Fos or ATF2 proteins [13], [14]. Binding sites for AP-1 transcription factor dimers are referred to as TRE (for TPA or tetradecanoylphorbol acetate response element). Such TREs confer transcription activation in response to JNK signaling to *cis*-linked genes. The JNK signaling system in the fruitfly is structurally and functionally similar to that of mammals. The *Drosophila* homolog of JNK is encoded by the gene *basket* (*bsk*) [15]. Basket is activated by the JNKK Hemipterous (Hep) which in turn responds to one of several JNKKKs [11], [16]. *Drosophila* Jun and Fos homologs are regulated by JNK phosphorylation [17], [18] and have the same DNA binding specificity as their mammalian counterparts.

Nrf2 is, like c-Jun, a leucine zipper transcription factor. The Nrf2 activating signaling pathway is turned on by diverse signals and stressors, some, but not all of which, also activate JNK [19], [20], [21]. Several compounds, including sulforaphane, oltipraz and lipoic acid, activate Nrf2 without causing evident cell stress. These drugs have therapeutic potential, for example in cancer chemoprevention [22]. Upon activation, Nrf2 dimerizes with the small leucine zipper protein Maf-S [23] and binds to its cognate promoter elements called AREs (for antioxidant response element). Genes thus regulated encode, among other proteins, redox regulators and phase II detoxification enzymes. Defects in the Nrf2 pathway cause stress sensitivity and Nrf2-deficient mouse mutants are predisposed to hyper-inflammatory diseases, cancer, neurodegeneration and metabolic dysfunction [20], [21].

The Nrf2 signaling system is functionally and structurally conserved in *Drosophila* [21], [24]. The fly homolog of Nrf2 is the Cap'n'collar splice form C (CncC) protein. CncC is regulated by interaction with an Nrf2-specific E3 enzyme, the *Drosophila* Keap1, and controls the expression of stress defense, detoxification and antioxidant genes reminiscent of the mammalian transcription factor. CncC function has been shown to confer resistance against acute oxidative stress, promote longevity, and regulate somatic stem cell maintenance in the *Drosophila* intestine [24], [25].

**Figure 1:**
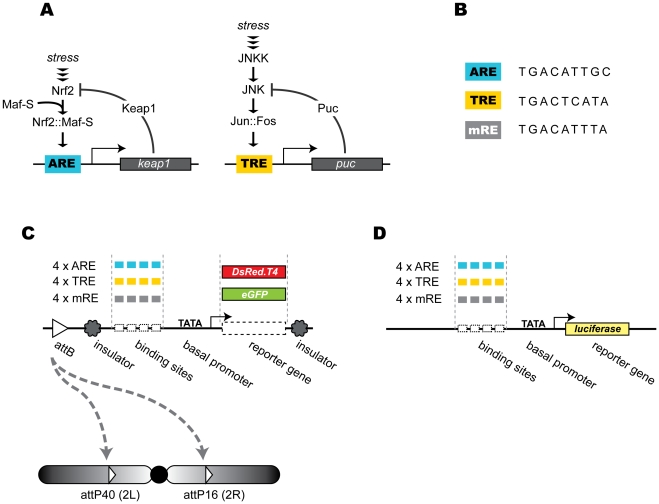
Design of ARE and TRE reporter genes. **A.** Simplified cartoons of stress signaling by the Nrf2 and JNK signaling pathways. Upon exposure to stress or cancer chemopreventive drugs, Nrf2 is relieved from repression by its cognate inhibitor Keap1. Nrf2 then dimerizes with Maf-S and binds to AREs of its target genes to activate their transcription. Activation of the *keap1* gene itself by Nrf2 engages a negative feedback loop that limits Nrf2 responses. The JNK cascade with the stress inducible JNKK and JNK activates the Jun::Fos dimer by phosphorylation. This leads to the activation of genes via Fos::Jun binding sites or TREs. Activation of the gene encoding the JNK phosphatase, Puckered causes negative feedback regulation. **B.** Sequence of the artificial TRE and ARE elements used for the construction of the Nrf2 and JNK reporters. The sequences correspond to optimal CncC/Maf and Jun/Fos binding sites as reported by Kerppola and Curran [29]. A mutant version of the response elements (mRE) that does not bind either Jun/Fos or Nrf2/Maf dimers was generated as a control construct. **C.** Modular design of *in vivo* reporter constructs. Each transgenic cassette carries an attB site for ΦC31 recombinase-catalyzed genomic integration, a minimal heat shock protein 70 (Hsp70) promoter (TATA) and eGFP or DsRed.T4 reporter gene driven by four head-to-tail concatenated copies of the ARE, TRE or mRE elements. The promoter-reporter cassettes are flanked by gypsy insulators to prevent ‘position effects’ of surrounding chromatin. Transgenic flies carry reporter transgenes at predetermined locations, attP 16 on the right arm of chromosome 2 and attP 40 on the left arm of the same chromosome. **D.** Design of reporters for cell culture experiments. In a similar design as shown in C, the firefly luciferase reporter gene was placed under a minimal heat shock protein 70 (Hsp70) promoter (TATA) and four head-to-tail concatenated copies of the ARE, TRE or mRE elements.

The transcriptional responses to JNK and Nrf2 signaling, are limited by negative feedback mechanisms mediated by the induction of specific inhibitors. The *Drosophila* JNK pathway, for example, stimulates the expression of the JNK phosphatase Puckered, which will eventually decrease the activity of the pathway by abrogating JNK kinase activity [26]. Nrf2 is subject to a similar feedback control involving the transcriptional upregulation of Keap1 (Fig. 1A) [27], [28].

The JNK and Nrf2 signaling pathways share several functional and molecular characteristics. The spectrum of extracellular signals and stressors that can activate JNK and Nrf2, respectively, is overlapping but not identical. Similarly, JNK activated transcription factors and Nrf2 have common, but also independent target genes. Whether there is significant cross-talk between JNK and Nrf2, and whether they function in parallel or synergistically in different cell types or organs has not systematically been investigated.

### Generation of *in vivo* and cell-based reporters

To elucidate the function and relationship of Nrf2 and JNK signaling, we designed a system of reporter genes for monitoring the functions of Nrf2 and JNK in intact organisms, tissues and cell culture. For the generation of *in vivo* reporter genes we used the previously described pelican plasmids as a starting point [2]. The key components of the new system are described below:

#### Synthetic transcription factor binding sites

Based on previously determined sequence preferences [29], [30], we designed DNA elements that would specifically bind either Nrf2/MafS dimers or Jun/Fos dimers (Fig. 1B). These oligonucleotides should therefore function as Nrf2-inducible ARE, or AP-1 responsive TRE elements, respectively. As a negative control, we generated a mutated version of the oligonucleotides, named mRE, which should not bind either Nrf2 or AP-1 as predicted based on the specificity of the respective mammalian homologs [29].

#### Reporter genes

Four head-to-tail concatenated copies of the ARE, TRE or mRE elements were placed upstream of a basal promoter to control the expression of either a green or red fluorescent protein, eGFP or DsRed.T4, respectively (Fig. 1C). These two proteins have distinct fluorescence spectra, which make it possible to assess their levels and distribution separately in the same sample. eGFP or DsRed.T4 have similar maturation time, facilitating the comparative analysis of Nrf2 and JNK signal response kinetics [2]. We will refer to these reporter constructs with names that indicate the nature of the promoter element and the reporter gene, as in ARE-red, TRE-green, mRE-green, etc.

#### Site-directed transgenesis with ΦC31

Conventional P-element-mediated transformation results in transgene insertion into random genomic loci. Consequently, the integrated gene can be exposed to variable position effects, which may seriously confound the interpretation of reporter experiments, especially when multiple different constructs are to be compared. In order to circumvent this problem, transgenic *Drosophila* lines carrying the ARE, TRE or mRE reporter genes in predetermined locations were generated using the ΦC31 recombinase mediated site directed transgenesis technique. This method utilizes the sequence-specific DNA recombinase of bacteriophage ΦC31 to target the transgenic construct carrying attB recombination sequence to a defined genomic position bearing a preplaced attP recipient site [7]. Two different docking sites, attP40 and attP16, on the left and the right arm, respectively, of the second chromosome were targeted for integration of the different reporter constructs (Fig. 1C). These genomic locations were chosen because previous studies had shown low basal and high induced expression of transgenes integrated at these sites [31]. As an additional safeguard against spurious or artifactual influences on reporter gene activity the response element-reporter cassette was flanked by gypsy insulators [31]. Several strains carrying different combinations of ARE, TRE, and mRE driving DsRed.T4 or eGFP reporters in either the 2 L or 2R integration site were generated (see Table 1 for nomenclature).

**Table 1:** Transgenic reporter fly lines.

Name	Promoter	Reporter	Location
ARE-green-2L	4×ARE	eGFP	attP40
TRE-red-2R	4×TRE	DsRed.T4	attP16
ARE-green-2R	4×ARE	eGFP	attP16
TRE-red-2L	4×TRE	DsRed.T4	attP40
ARE-red-2L	4×ARE	DsRed.T4	attP40
TRE-green-2R	4×TRE	eGFP	attP16
mRE- red-2L	4×mRE	DsRed.T4	attP40

#### Reporters for cell culture experiments

As a complement to the *in vivo* reporters in transgenic flies, we generated matching cell-based reporters, namely ARE-fluc, TRE-fluc and mRE-fluc that carry the firefly luciferase gene under the control of the same synthetic Nrf2 and AP-1 binding sites (Fig. 1D). These *in vitro* reporters can be used to carry out, among other experiments, large-scale screens for genetic regulators and chemical modulators of CncC and JNK pathways.

### Functional Characterization

#### *In vivo* reporters

**Figure 2:**
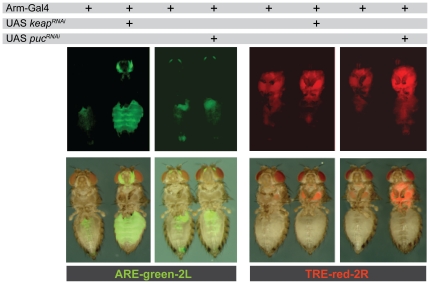
Transgenic ARE reporters and TRE reporters are specifically responsive to Nrf2 and JNK signaling respectively. Loss of dKeap1 function specifically activates Nrf2 activity *in vivo* [24]. Consequently, expressing a Keap1-specific RNAi under the control of the ubiquitously active arm-Gal4 driver activated the ARE-green reporter, but not the TRE-red reporter. Conversely, arm-Gal4 driven expression of RNAi targeting Puc, the negative regulator of JNK signaling, stimulated TRE-red reporter activity but did not affect the ARE-green reporter. The upper panels show red and green fluorescence in adult 5-day-old female flies. These fluorescence images were superimposed onto the bright-field images of the same flies, as shown in the lower panels. 15 to 20 flies were analyzed for each genotype and all showed similar results. The images shown show representative, randomly selected specimens.

The readout of fluorescent ARE or TRE reporters can be monitored in intact flies using a UV equipped stereo microscope. Fig. 2 shows that in standard non-stressed conditions both ARE and TRE-dependent reporters have measureable basal levels of activity. Interestingly, the tissue distribution of the ARE and TRE dependent signals differs, with the ARE reporter showing prominent activity in the abdomen and the antennae, whereas TRE activity was strongest in the thorax. This evident tissue specificity is independent of the genomic integration site. A qualitative analysis shows that the activity pattern is maintained regardless of whether the reporters reside in the attP16 or attP40 location (Fig. S1). The ARE and TRE reporters will be instrumental to dissect tissue and organ-specific functions of JNK and Nrf2 signaling.

In order to test the inducibility and specificity of the ARE and TRE reporters in their respective transgenic fly lines, we assessed the effect of activating the Nrf2 and JNK pathways genetically. To this end, the specific inhibitors of JNK or Nrf2 signaling, Puc and dKeap1, respectively, were removed by expression of corresponding RNAis under the control of the ubiquitously active armadillo Gal4 driver. The resulting changes in reporter gene activity were assessed at the whole animal level by observation under a stereo microscope (Fig. 2). Knockdown of dKeap1 specifically activated the ARE-green reporter, but not the TRE-red reporter. Conversely, removal of Puc activated TRE-red reporter in flies (Fig. 2), but left ARE-green unaffected.

**Figure 3:**
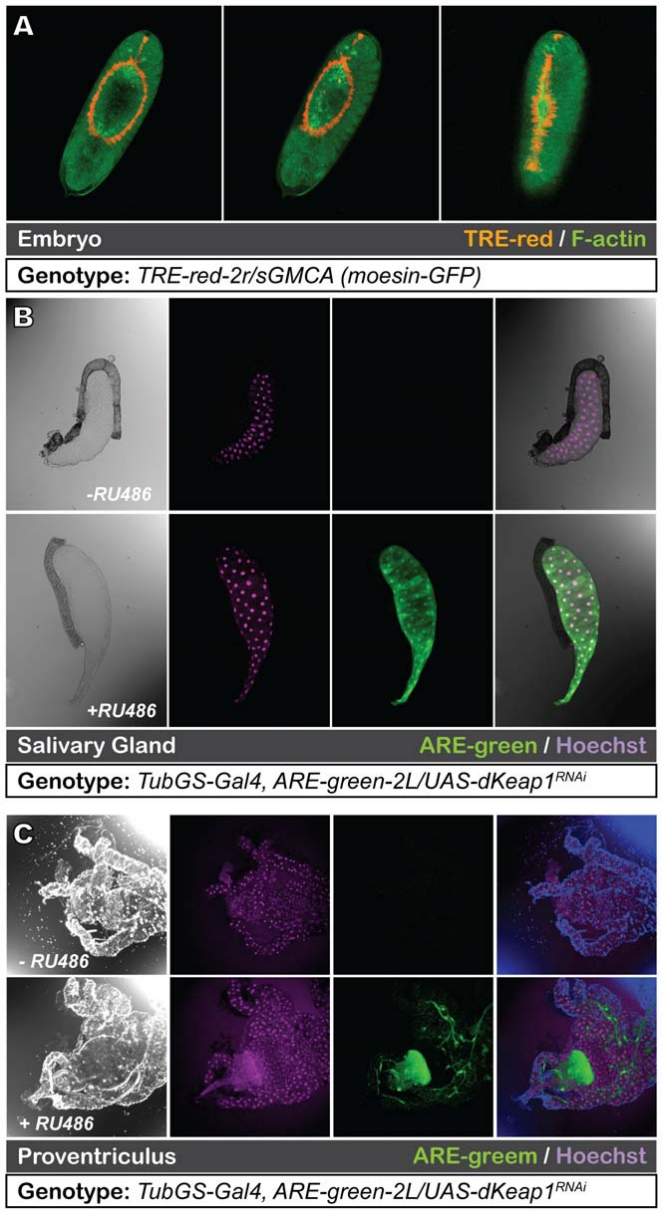
ARE and TRE reporters faithfully reproduce known patterns of JNK and Nrf2 signaling. A. The TRE-red reporter marks the leading edge cells of stage 13 to 15 embryos where JNK is known to be active during dorsal closure. In addition to the TRE-red-2R reporter, the embryos expressed a moesin GFP fusion protein (sGMCA [47]) to visualize the actin cytoskeleton (green). Three embryos at progressively later stages of dorsal closure are shown. B. ARE-green reporter activity was detected in different larval tissues in which Nrf2 function has previously been reported [24]. In 3^rd^ instar larval salivary glands Nrf2 reporter activity was undetectable at basal levels (top panel, genotype TubGSGal4, ARE-green-2L/UAS-dKeap1^RNAi^, no RU 486 treatment), but was prominent after knockdown of Keap1 expression (same genotype as above, but keap1^RNAi^ expression was induced by RU486). The nuclei are stained with Hoechst (purple). The left panels show a micrograph of the salivary glands. C. Cells in the proventriculus of the digestive tract show prominent Nrf2 activity after Keap1 knockdown. Conditions and genotypes are as described in panel B.

Confirming the fidelity of the *in vivo* reporter lines, we found that they mark tissues in which the JNK or Nrf2 signaling is known to be active (Fig. 3). For example, the TRE reporter strongly labels cells in the leading edge of the embryonic epidermis during dorsal closure, a morphogenetic process that requires developmentally regulated JNK mediated gene activation in this location [32], [33], [34], [35]. High inducible Nrf2 target gene and CncC mRNA expression, has been described in the proventriulus, midgut and salivary glands of *Drosophila* larvae [24]. This activity pattern can be recapitulated in larvae expressing *keap1^RNAi^* (used in Sykiotis and Bohmann, 2008 [24]), as visualized with the ARE-green reporter (Fig. 3). These results show that the ARE and TRE reporters can be used to follow the *in vivo* CncC and JNK activity of *Drosophila* at various stages of development and during different physiological processes.

#### *In vitro* reporters

**Figure 4:**
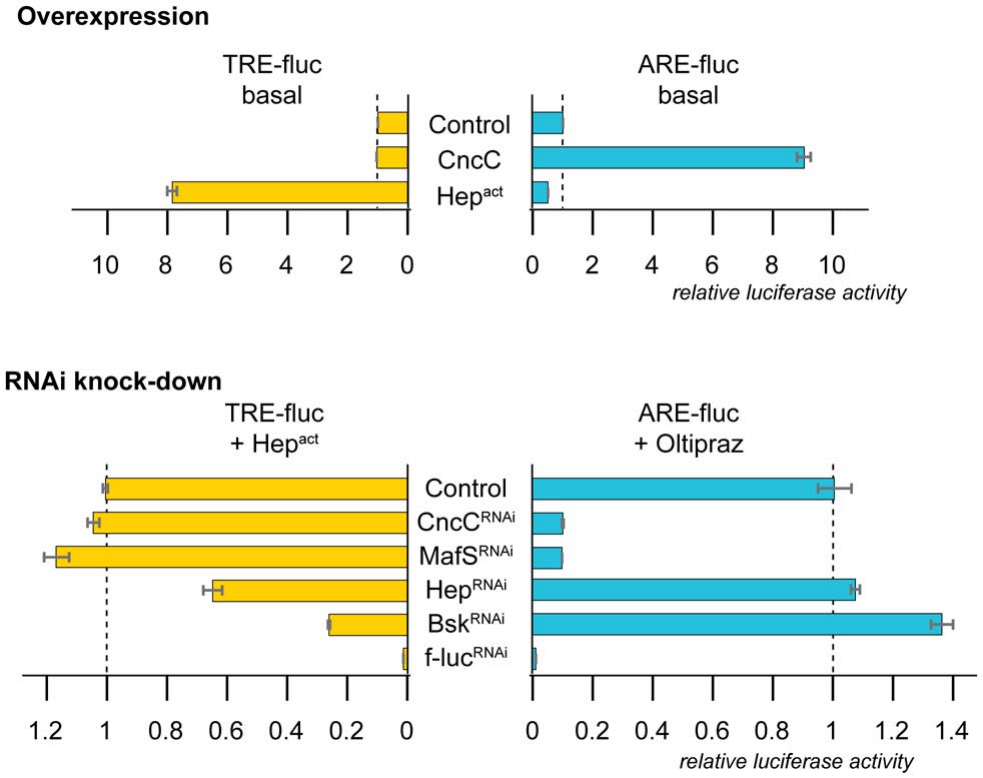
Signal specificity of cell-based reporters. A. Artificial stimulation of JNK and Nrf2 signaling in S2 cells specifically activates the TRE-fluc or ARE-fluc reporter, respectively. Expression of a constitutively active mutant of *Drosophila* JNKK (Hep^act^) activated a co-transfected TRE-fluc reporter, but not an ARE-fluc reporter. Conversely, overexpression of CncC increased the activity of the ARE and not the TRE reporter. Firefly luciferase activity was normalized using a cotransfected renilla luciferase construct (Act5C rluc) as a reference. The activity under control conditions (reporters co-transfected with pAct-Gal4) was set to 1. B. Signal-dependent activation of TRE and ARE reporters specifically requires JNK or Nrf2 pathway components. S2 cells were transfected with TRE-fluc reporters and JNK activity was stimulated by co-expression of constitutively active *Drosophila* JNKK (Hep^act^) as indicated. Cells transfected with the ARE-fluc reporter were treated with the specific Nrf2 activator Oltipraz. dsRNA mediated knockdown of CncC, or MafS compromised only Oltipraz-induced ARE-fluc activity. Conversely, dsRNA against Hep (JNKK) and Bsk (JNK) only reduced TRE activity. Firefly luciferase activity was normalized to renilla luciferase activity driven by Act5C promoter. The activity of the activated reporters under control conditions was set to 1. Error bars indicate standard deviation of triplicate measurements.

Complementing the *in vivo* experiments described above, cell culture assays were designed to test the specificity of the ARE and TRE luciferase reporters in S2 cells. The JNK pathway can specifically be activated in this system by transient expression of a constitutively active version of JNKK (Hep^act^, in which regulatory phosphorylation sites, the serine and threonine residues at positions 326 and 330, were replaced with aspartic acid), a kinase that selectively phosphorylates JNK and causes its robust activation [36], [37]. Under these conditions, AP-1 activity, as measured by the TRE-fluc reporter and luciferase assays, increases strongly. In contrast, the ARE-fluc reporters did not respond in parallel experiments, documenting the specificity of the assay system. Conversely, when the *Drosophila* Nrf2 pathway was genetically activated, by overexpression of CncC, enhanced ARE but not TRE activity was observed (Fig. 4a)

In loss-of-function experiments, the activities of ARE-fluc and TRE-fluc reporters were specifically downregulated by dsRNA-mediated knock-down of CncC and Bsk (Fig. 4b). These results show that the ARE-luc reporter is specific to CncC signaling, whereas the TRE-fluc is specific to JNK activity in S2 cells. Taken together, the cell-based and *in vivo* studies showed that the genetic modulation of one pathway does not affect the activity of the reporter for the other pathway. Therefore, the ARE and TRE reporters can be reliably used to monitor CncC and JNK activity both *in vivo* and in cell-based experiments.

### Comparative analysis of stress JNK and Nrf2 responses in adult flies and cell culture

Due to the defined genomic integration sites of the *in vivo* reporters and the complementary spectral characteristics of eGFP and dsRedT4, it is possible to generate fly stocks carrying two different reporters that can be tracked separately. For example, we constructed a recombinant *Drosophila* line that carries 2 copies of ARE-green and 2 copies of TRE-red reporters at the attP40 and attP16 sites respectively, on the second chromosome. In these flies, JNK signaling can be tracked as red fluorescence whereas Nrf2 activation elicits a green signal.

**Figure 5:**
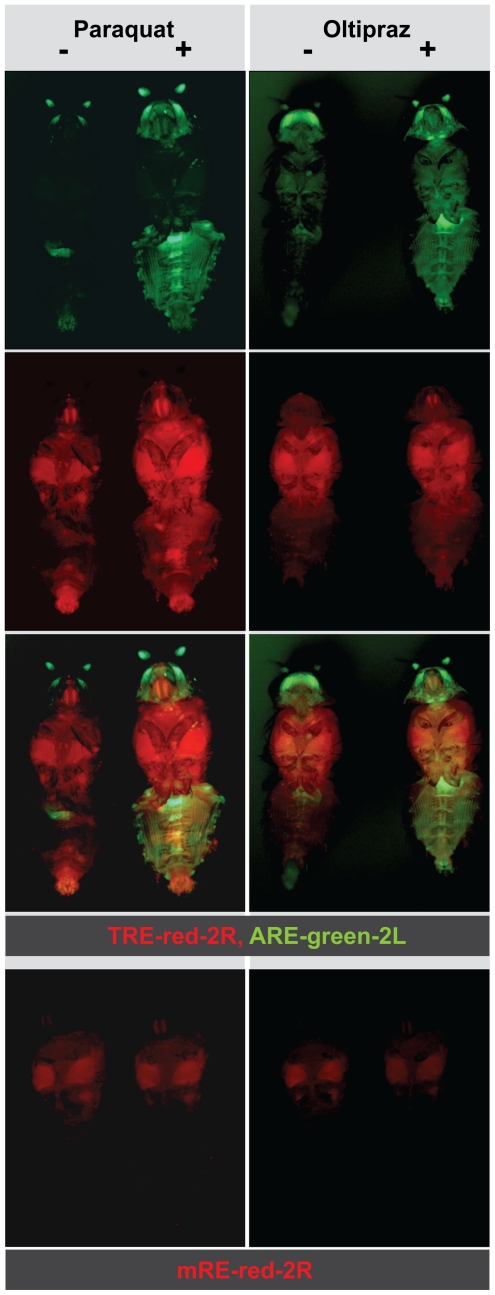
Simultaneous measurement of Nrf2 and JNK responses *in vivo*. Exposure of flies that carry two copies each of both the TRE-red-2R and the ARE-green-2L reporters to the oxidative stressor paraquat increased both ARE and TRE activities, however with different spatial specificity. Oral application of the cancer chemopreventive drug oltipraz, on the other hand, only induced ARE and not TRE activity. The upper and the middle panels show green and red fluorescence separately in the same flies. These green and red fluorescence images are merged in the lower panel. The control food for oltipraz treatment was supplemented with 1% DMSO, the solvent used for oltipraz. The control reporter stock mRE-red-2L did not respond to either paraquat or oltipraz.

Using these double reporter flies, JNK and Nrf2 responses to different insults, as well as the temporal and spatial characteristics of their induction, can readily be compared in the intact organism. Fig. 5 shows an example for such an analysis. Both JNK and Nrf2 activity were markedly elevated upon exposure of the double reporter flies to the oxidative stressor paraquat, a free radical generator. Similar effects were observed in response to treatment with hydrogen peroxide (an intracellular reactive oxygen species), sodium arsenite (a heavy metal), or DEM (diethyl maleate, a glutathione-depleting agent) (data not shown). In contrast, exposure to Oltipraz, a cancer chemopreventive drug known to mediate its salutary effects by boosting Nrf2 activity [38], [39], raised the ARE-GFP signal, but had no effect on the activity of the TRE reporter.

**Figure 6:**
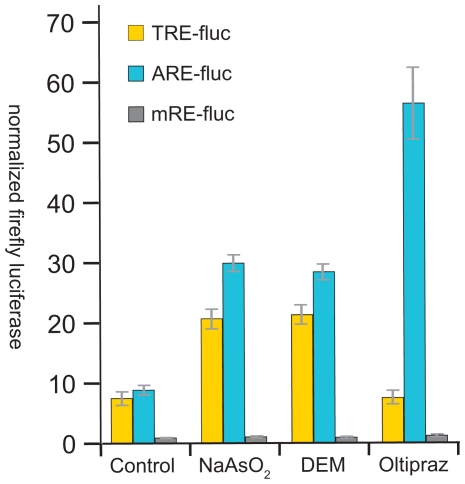
Stimulation of cell-based reporters by sulforaphane, oltipraz and stressors. S2 cells transiently transfected with TRE-fluc, ARE-fluc or mRE-fluc plasmids, as indicated, were exposed to sodium arsenite (NaAsO_2_), Diethyl Maleate (DEM) or oltipraz. The firefly luciferase activity was measured 24 hrs after drug treatment and normalized to renilla luciferase activity expressed from a co-transfected Act5C renilla luciferase construct. Note that both TRE and ARE reporters respond to the stressors, Arsenite and DEM, but only ARE-fluc responds to Oltipraz. The mRE reporter responds neither to oltipraz nor to the stressors. The activity of mRE-fluc reporter under control conditions was set to 1. Error bars indicate standard deviation of triplicate measurements.

To test whether signal specific pathway activation, as documented *in vivo*, can also be observed in S2 cultures, experiments using the ARE, TRE and mRE-fluc reporters were conducted. We found that Nrf2 activity, as measured by a transiently transfected ARE-fluc reporter, was selectively induced by treatment of S2 cells with Oltpraz or Sulforaphane (Fig. 6 and data not shown). TRE-fluc does not respond to these drugs. In contrast, general stressors DEM and arsenite stimulate both the ARE and the TRE reporter (Fig. 6). The mRE reporter is not affected by any of these drugs. These experiments show the utility of the reporter system for studying CncC and JNK responses in cell culture, allowing further molecular and biochemical characterization of different stressful or pharmacological activators and inhibitors.

## Discussion

The integrated reporter system described in this report meets its intended specifications:

Nrf2 and AP-1 activity can be measured accurately, specifically and sensitively *in vitro*. The *in vivo* reporter lines permit observation of spatial patterns of JNK and Nrf2 signaling and a semi-quantitative assessment of changes in signaling strength brought about by genetic or environmental factors.

Known or predicted activity patterns, such as the activation of JNK in the leading edge of the dorsally closing embryonic epidermis, or the Nrf2 activity in the digestive tract, are reproduced.

The two insertion sites, attP40 and attP16, were chosen because they were reported to confer little base level activity onto inserted transgenes. Consistently, we see very low background activity with the mRE control reporter. Swapping insertion points does not change the observed pattern of activity, indicating that the respective chromosomal environments have minimal effect on the reporters and that results from genes inserted in attP16 and attP40 can be directly compared.

Responses are specific: JNK and Nrf2 signaling activates only their respective reporters without “spillover” into the other pathway.

Compared to existing P-element based systems, the reporters described here offer several advantages: The *Drosophila* reporter strains carry DsRed.T4 or eGFP constructs in one of two defined chromosomal positions that were selected for low background activity and neutral responses to the different stress treatments tested so far. This standardized chromosomal integration makes it possible to directly compare the activities of different reporters. This is useful, for example, when comparing ARE or TRE reporters with the mutant version (mRE) that serves as a background control.

The two integration sites chosen are on the two arms of the second chromosome. Pairs of red and green reporters can thus be easily combined, generating stable dual reporter stocks. In this manner, Nrf2 and JNK activity can be tracked independently in the same specimen. It is also possible to combine ARE or TRE reporters with tissue or cell type specific red or green markers or with the control mRE vector. The similar maturation time of eGFP and DsRed.T4 further assists this comparative analysis.

The plasmid constructions are designed in a way that the system can easily accommodate other synthetic or natural promoter or enhancer sequences. Using straightforward recombinant DNA and *Drosophila* transgene technology, the system can be adapted to track other signaling pathways or transcription factors.

Luciferase versions of the same reporter genes show the regulatory characteristics and signal responsiveness in S2 cell transfections that was predicted from the behavior of the *in vivo* reporters. For example, both *in vivo* and *in vitro* assays document that Nrf2 and JNK responses are regulated by independent upstream pathways. The S2 cell system can be used for medium or high throughput RNAi or drug screens. The integrated reporter system allows rapid *in vivo* validation of genes or mechanisms pinpointed by cell based screens.

While JNK, as well as Nrf2, reporters have been constructed for mammals, there are no reports of a comprehensive, standardized and directly comparable system like the one we have generated for *Drosophila*. For example, Tsai et al [40] used a mammalian reporter that contains an ARE-driven luciferase gene. However, this construct also contains nucleotides that confer JNK specificity. Huang et al [41] used transgenic mice carrying a TRE-luciferase reporter that is responsive to JNK signaling.

## Materials and Methods

### Plasmids and fly lines

Synthetic double stranded oligonucleotides, carrying four high affinity AP-1 or Nrf2 binding sites or a mutant control (4×ARE, 4×TRE and 4×mRE, see Fig. 1B) were inserted into modified pGL3-Basic, a plasmid that includes *Drosophila* Hsp70 minimal promoter driving the expression of firefly luciferase gene [42] (kindly provided by Dr. Michael Boutros) to generate cell-based reporter plasmids. The *in vivo* reporter plasmids were generated in multiple steps. 4×ARE was transferred into the pGreen H-Pelican vector to generate pGreen-ARE. The attB sequence from piB-GFP [43] plasmid was inserted into pBSKS vector to generate pBSKS-attB plasmid. The ARE-eGFP cassette along with the flanking gypsy insulators was amplified by PCR and inserted into pBSKS-attB to generate pB-ARE-green. The 4×ARE promoter element and eGFP reporter gene of this plasmid was replaced with other promoter elements (e.g., 4×TRE, 4×mRE) and other reporter genes (e.g., DsRed.T4), respectively, to generate plasmids with different combinations of promoters and reporters.

ΦC31 recombinase-mediated site-directed transgenesis was used to generate transgenic fly lines (service provided by Genetic Services Inc, MA). Individual reporter fly lines were then recombined with other reporter fly lines to facilitate the simultaneous study of two different promoter elements in the same organism. For overexpression or RNAi experiments in which genetic effects on reporter activity were analyzed, the reporter lines were also recombined with different driver lines (e.g, arm-Gal4, tubGS-Gal4).

Plasmids maps and sequence files are included as Text S1.

### Calcium phosphate transfection

*Drosophila* S2 cells were transiently transfected with firefly luciferase reporter constructs using the calcium phosphate method. A co-transfected actin-driven renilla luciferase reporter plasmid (pAct-RL) [44] (kindly provided by Dr. Willis Li) served as an internal reference for normalization. S2 cells grown in M3+BPYE (Sigma-Aldrich, St. Louis, MO) with 10% FBS and supplemented with penicillin and streptomycin were seeded at 2×10^6^ cells/ml concentration 6 hours before transfection. The transfection mix was prepared by combining Solution A (0.25 M CaCl_2_, 0.5 µg/ml of the firefly luciferase reporter construct, 0.2 µg/ml pAct-RL, 1.3 µg/ml salmon sperm DNA) and Solution B (2×HBS: 50 mM HEPES-KOH, 1.5 mm Na_2_HPO_4_, 280 mM NaCl, pH 7.1). Solution A was slowly added drop wise to an equal volume of Solution B with continuous vortexing and the mixture was incubated at room temperature for 30 minutes to allow formation of a fine precipitate. The mixture was briefly vortexed before adding it drop wise to the seeded cells with constant swirling. 1 ml of seeded S2 cells was incubated with 0.5 ml of transfection mix (for a total of 250 ng ARE-fluc/TRE-fluc/mRE-fluc, 100 ng of pAct-RL, 650 ng salmon sperm DNA per well) at 25°C for 16 hrs before the calcium phosphate solution was aspirated, cells were washed with 1×PBS and fresh culture medium was added. The Solution A for CncC and Hep^act^ over expression experiments contained 0.25 M CaCl_2_, 0.5 µg/ml of the firefly luciferase reporter construct, 0.2 µg/ml pAct-RL, 0.5 µg/ml pAct-Gal4 and 1 µg/ml pUAST-CncC or pUAST-Hep^act^. pUAST-CncC and pUAST-Hep^act^ were replaced with 1 µg/ml salmon sperm DNA in the control group of these experiments.

### Chemical and drug treatments

To study the effect of different oxidative stressors on reporter gene expression in transgenic flies, 1 week old flies that were mated for one day and then separated into males and females, were exposed to different oxidative stressors. The animals were starved for 2 hours and then fed a solution of 5% sucrose ±20 mM Paraquat (Sigma-Aldrich, St. Louis, MO). To examine the effect of oltipraz on reporter flies, similarly collected reporter flies were fed food supplemented with 1 mM oltipraz (LKT Laboratories Inc., St. Paul, MN) for 48 hours. 15–20 flies were used in each group for these experiments and 3–5 representative flies were chosen randomly for imaging. Similar results were seen in multiple experiments. Western blot for reporter proteins on pools of flies also confirmed the induction of reporter transgenes with different treatments (data not shown).

To assess the effect of different oxidative stressors and chemicals on the cell-based reporters S2 cells were transiently transfected with the reporter plasmids by the calcium phosphate method. 8 hours after the PBS wash and medium change, the transfected cells were transferred to 96-well plates and treated with 25 µM oltipraz, 100 µM DEM, 100 µM NaAsO_2_ (J.T.Baker (Phillipsburg, NJ)) and were incubated at 25°C for 24 hrs.

### dsRNA synthesis and treatment

dsRNAs were synthesized and purified following the protocol provided by Sandra Steinbrink and Michael Boutros [45] using ‘T7 RiboMAX Express RNAi System’ kit (Promega) and ‘RNeasy Kit’ (QIAGEN). Predesigned RNA probes were used to knock-down CncC, Maf-S, Hep and Bsk (Table S1) whereas dsRNAs against GFP and firefly luciferase were designed using E-RNAi webservice [46]. S2 cells (1×10^6^ cells/ml concentration) were bathed with dsRNAs in 12-well plates (8 µg dsRNA/well) as described [45]. dsRNA-treated cells were transfected with luciferase reporter plasmids by calcium phosphate method 3 days after dsRNA bathing.

### Luciferase assay

The activities of cell-based reporters were measured by ‘Dual Glo Luciferase Assay System’ kit (Promega). 75 µl of transfected S2 cells (either untreated or treated with dsRNA and/or chemicals) were transferred to wells of 96-well luciferase assay plates and luciferase assay was carried out in triplicate following the protocol provided by the supplier.

### Microscopy

To study JNK activity during dorsal closure TRE-red-2R/SGMCA stage 13 embryos were collected on apple plates and were dechorionated manually on double sided tape. Live embryos were mounted in PCTFE oil (HPC#27∶HPC#200 = 1∶1, from Halocarbon Products Corp., NJ). Confocal images were collected using a Leica TCS SP5 system and were processed using Adobe Photoshop.

To study CncC activity tubGS-Gal4, ARE-green-2L/UAS-dKeap1^RNAi^; UAS-dKeap1^RNAi^/+ stage 2 larvae were treated with inducer RU486 (Cayman Chemical, Ann Arbor, MI) by addition of 2 mg/ml RU486 in 50% ethanol to the food. The control group of the same genotype was treated with 50% ethanol. Both the control and treated larvae were dissected at stage 3 in PBS and salivary glands and guts were fixed at room temperature for 30 minutes in 100 mM glutamic acid, 25 mM KCl, 20 mM MgSO_4_, 4 mM sodium phosphate, 1 mM MgCl_2_, and 4% formaldehyde. DNA was stained by Hoechst. Confocal images were collected and processed as above.

## Supporting Information

Figure S1 **Insertion site or type of reporter gene do not affect** ***in vivo*** **ARE activity.** ARE-red-2L, ARE-green-2R double Nrf2 reporter flies were exposed to DEM stress. Both basal level and DEM-induced activities of ARE reporters at two different sites, attP40 (2L) and attP16 (2R), containing different reporter genes (eGFP and DsRedT4) were similar.
(TIF)

Text S1 **Plasmid maps and sequences of** ***in vivo*** **reporters.** The maps and sequences for plasmids pB-ARE-Green, pB-ARE-Red, pB-TRE-Green, pB-TRE-Red and pB-mRE-Red are shown. These plasmids were inserted in attP40 and attP16 sites to generate transgenic reporter fly lines.
(PDF)

Table S1 **Primers used to generate amplicons for dsRNA synthesis.**
(DOC)
