# Supplementary figures and images for: A Versatile ΦC31 Based Reporter System for Measuring AP-1 and Nrf2 Signaling in Drosophila and in Tissue Culture
Source: PLoS One. 2012 Apr 11;7(4):e34063. doi: 10.1371/journal.pone.0034063 (PMC3324472; doi:10.1371/journal.pone.0034063)

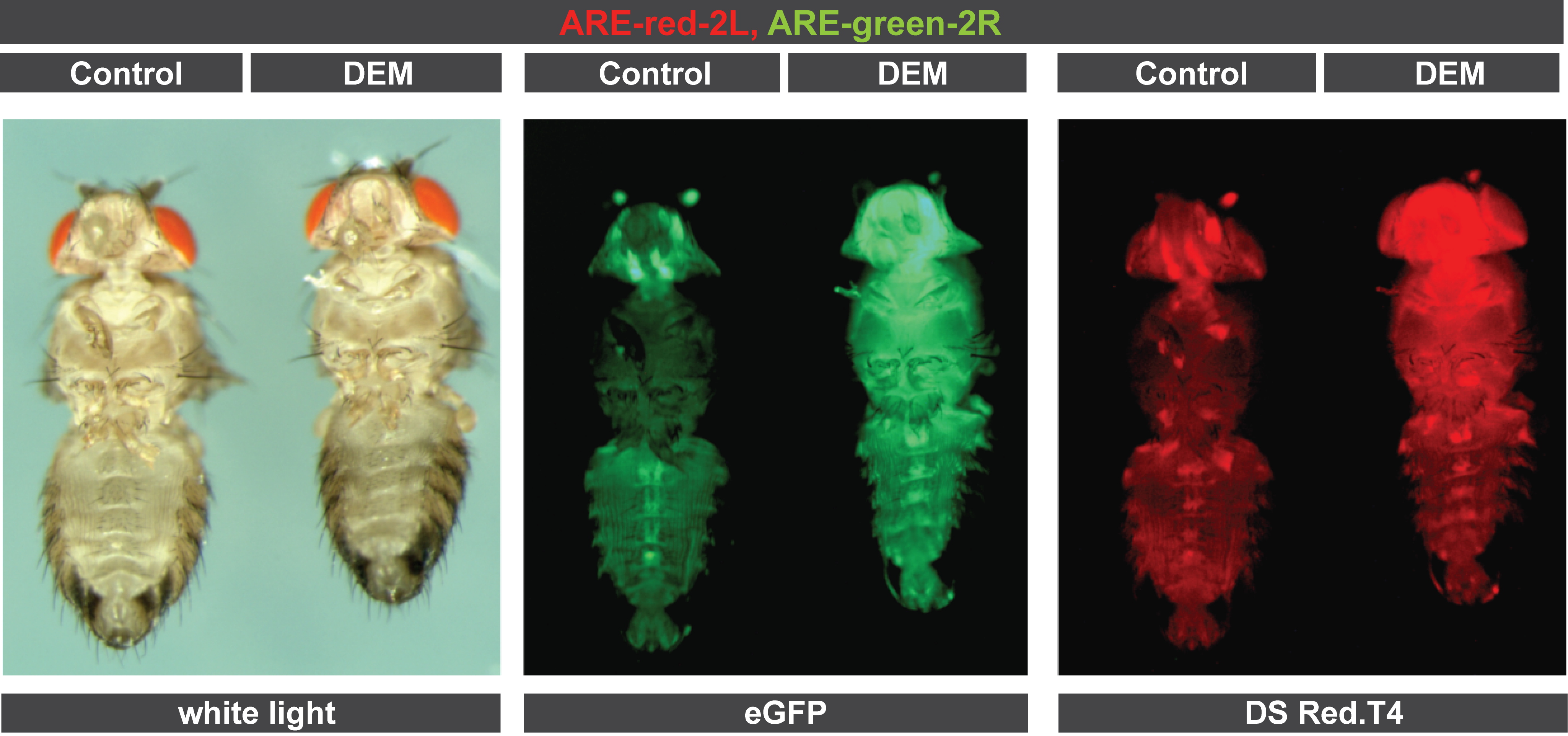

Supplement: Figure S1 — Insertion site or type of reporter gene do not affect in vivo ARE activity. ARE-red-2L, ARE-green-2R double Nrf2 reporter flies were exposed to DEM stress. Both basal level and DEM-induced activities of ARE reporters at two different sites, attP40 (2L) and attP16 (2R), containing different reporter genes (eGFP and DsRedT4) were similar. (TIF) [file pone.0034063.s001.tif]
