# Supplementary material for: A Versatile ΦC31 Based Reporter System for Measuring AP-1 and Nrf2 Signaling in Drosophila and in Tissue Culture
Source: PLoS One. 2012 Apr 11;7(4):e34063. doi: 10.1371/journal.pone.0034063 (PMC3324472; doi:10.1371/journal.pone.0034063)
Supplement: Text S1 — Plasmid maps and sequences of in vivo reporters. The maps and sequences for plasmids pB-ARE-Green, pB-ARE-Red, pB-TRE-Green, pB-TRE-Red and pB-mRE-Red are shown. These plasmids were inserted in attP40 and attP16 sites to generate transgenic reporter fly lines. (PDF) [file pone.0034063.s002.pdf]

pB-ARE-Green

Plasmid backbone:pBluescript II KS(+)
attB: 2208-2516
gypsy insulator: 2532-2927; 4222-4607
4X ARE: 2937-3016
eGFP: 3023-4192
mini-white: 5095-7967

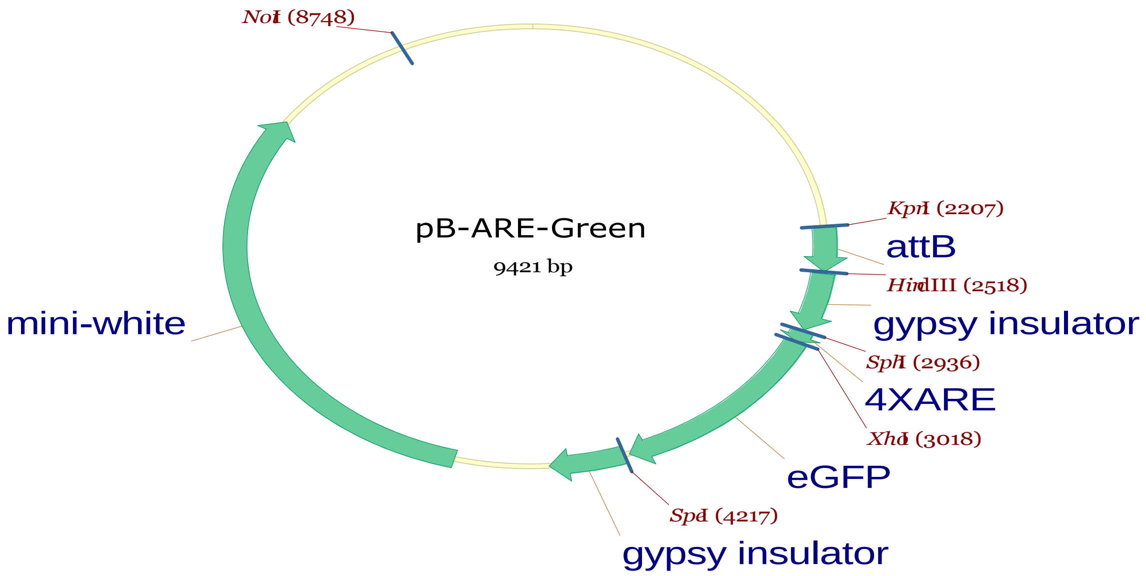

gtggcacttttcggggaaatgtgcgcggaacccctatttgtttatttttctaaatacattcaaatatgtatccgctcatg
agacaataaccctgataaatgcttcaataatattgaaaaaggaagagtatgagtattcaacatttccgtgtcgccc
ttattcccttttttgcggcattttgccttcctgtttttgctcacccagaaacgctggtgaaagtaaaagatgctgaagat
cagttgggtgcacgagtgggttacatcgaactggatctcaacagcggtaagatccttgagagttttcgccccgaa
gaacgttttccaatgatgagcacttttaaagttctgctatgtggcgcggtattatcccgtattgacgccgggcaaga
gcaactcggtcgccgcatacactattctcagaatgacttggttgagtactcaccagtcacagaaaagcatcttac
ggatggcatgacagtaagagaattatgcagtgctgccataaccatgagtgataacactgcggccaacttacttct
gacaacgatcggaggaccgaaggagctaaccgcttttttgcacaacatgggggatcatgtaactcgccttgatc
gttgggaaccggagctgaatgaagccataccaaacgacgagcgtgacaccacgatgcctgtagcaatggca
acaacgttgcgcaaactattaactggcgaactacttactctagcttcccggcaacaattaatagactggatggag
gcggataaagttgcaggaccacttctgcgctcggcccttccggctggctggtttattgctgataaatctggagccg
gtgagcgtgggtctcgcggtatcattgcagcactggggccagatggtaagccctcccgtatcgtagttatctacac
gacggggagtcaggcaactatggatgaacgaaatagacagatcgctgagataggtgcctcactgattaagcat
tggtaactgtcagaccaagtttactcatatatactttagattgatttaaaacttcatttttaatttaaaaggatctaggtg
aagatcctttttgataatctcatgaccaaaatcccttaacgtgagttttcgttccactgagcgtcagaccccgtaga
aaagatcaaaggatcttcttgagatcctttttttctgcgcgtaatctgctgcttgcaaacaaaaaaaccaccgctac
cagcggtggtttgtttgccggatcaagagctaccaactctttttccgaaggtaactggcttcagcagagcgcagat
accaaatactgtccttctagtgtagccgtagttaggccaccacttcaagaactctgtagcaccgcctacatacctc
gctctgctaatcctgttaccagtggctgctgccagtggcgataagtcgtgtcttaccgggttggactcaagacgat
agttaccggataaggcgcagcggtcgggctgaacggggggttcgtgcacacagcccagcttggagcgaacg
acctacaccgaactgagatacctacagcgtgagctatgagaaagcgccacgcttcccgaagggagaaaggc
ggacaggtatccggtaagcggcagggtcggaacaggagagcgcacgagggagcttccagggggaaacgc
ctggtatctttatagtcctgtcgggtttcgccacctctgacttgagcgtcgatttttgtgatgctcgtcaggggggcgg
agcctatggaaaaacgccagcaacgcggcctttttacggttcctggccttttgctggccttttgctcacatgttctttc
ctgcgttatcccctgattctgtggataaccgtattaccgcctttgagtgagctgataccgctcgccgcagccgaac
gaccgagcgcagcgagtcagtgagcgaggaagcggaagagcgcccaatacgcaaaccgcctctccccgc

gcgttggccgattcattaatgcagctggcacgacaggtttcccgactggaaagcgggcagtgagcgcaacgca
attaatgtgagttagctcactcattaggcaccccaggctttacactttatgcttccggctcgtatgttgtgtggaattgt
gagcggataacaatttcacacaggaaacagctatgaccatgattacgccaagcgcgcaattaaccctcactaa
agggaacaaaagctgggtaccgggccccccctcgacgatgtaggtcacggtctcgaagccgcggtgcgggt
gccagggcgtgcccttgggctccccgggcgcgtactccacctcacccatctggtccatcatgatgaacgggtcg
aggtggcggtagttgatcccggcgaacgcgcggcgcaccgggaagccctcgccctcgaaaccgctgggcgc
ggtggtcacggtgagcacgggacgtgcgacggcgtcggcgggtgcggatacgcggggcagcgtcagcgggt
tctcgacggtcacggcgggcatgtcgaggtcgacggtatcgataagcttgggctgcatcacgtaataagtgtgc
gttgaatttattcgcaaaaacattgcatattttcggcaaagtaaaattttgttgcataccttatcaaaaaataagtgct
gcatactttttagagaaaccaaataatttttattgcatacccgtttttaataaaatacattgcataccctcttttaataa
aaaatattgcatactttgacgaaacaaattttcgttgcatacccaataaaagattatatattgcatacccgtttttaat
aaaatacattgcataccctcttttaataaaaaatattgcatacgttgacgaaacaaattttcgttgcatacccaataa
aagattattatattgcataccttttcttgccataccatttagccgatcaattgtgctcggcaacagcatgcgatttctgc
aatgtcatcgtgatttctgcaatgtcatcgtgatttctgcaatgtcatcgtgatttctgcaatgtcatcgtctcgaggag
cgccggagtataaatagaggcgcttcgtctacggagcgacaattcaattcaaacaagcaaagtgaacacgtc
gctaagcgaaagctaagcaaataaacaagcgcagctgaacaagctaaacaatctgcagccaagctgatcct
ctagggtacgcgctagagtcgagaggcctgtttaaacgatccaccggtcgccaccatggtgagcaagggcga
ggagctgttcaccggggtggtgcccatcctggtcgagctggacggcgacgtaaacggccacaagttcagcgtg
tccggcgagggcgagggcgatgccacctacggcaagctgaccctgaagttcatctgcaccaccggcaagctg
cccgtgccctggcccaccctcgtgaccaccctgacctacggcgtgcagtgcttcagccgctaccccgaccacat
gaagcagcacgacttcttcaagtccgccatgcccgaaggctacgtccaggagcgcaccatcttcttcaaggac
gacggcaactacaagacccgcgccgaggtgaagttcgagggcgacaccctggtgaaccgcatcgagctga
agggcatcgacttcaaggaggacggcaacatcctggggcacaagctggagtacaactacaacagccacaa
cgtctatatcatggccgacaagcagaagaacggcatcaaggtgaacttcaagatccgccacaacatcgagga
cggcagcgtgcagctcgccgaccactaccagcagaacacccccatcggcgacggccccgtgctgctgcccg
acaaccactacctgagcacccagtccgccctgagcaaagaccccaacgagaagcgcgatcacatggtcctg
ctggagttcgtgaccgccgccgggatcactctcggcatggacgagctgtacaagtaaagcggccggccgcga
ctctagatcataatcagccataccacatttgtagaggttttacttgctttaaaaaacctcccacacctccccctgaa
cctgaaacataaaatgaatgcaattgttgttgttaacttgtttattgcagcttataatggttacaaataaagcaatagc
atcacaaatttcacaaataaagcatttttttcactgcattctagttgtggtttgtccaaactcatcaatgtatcttaagg
cgtaaattgtaagcgttaatgactagtcacgtaataagtgtgcgttgaatttattcgcaaaaacattgcatattttcgg
caaagtaaaattttgttgcataccttatcaaaaaataagtgctgcatactttttagagaaaccaaataatttttattgc
atacccgtttttaataaaatacattgcataccctcttttaataaaaaatattgcatactttgacgaaacaaattttcgtt
gcatacccaataaaagattatatattgcatacccgtttttaataaaatacattgcataccctcttttaataaaaaata
ttgcatacgttgacgaaacaaattttcgttgcatacccaataaaagattatatattgcataccttttcttgccatacca
tttagccgatcaattctagtatgtatgtaagttaataaaaccctttttttggagaatgtagatttaaaaaaacatattttttt
tttatttttttactgcactggacatcattgaacttatctgatcagttttaaatttacttcgatccaagggtatttgaagtacc
aggttctttcgattacctctcactcaaaatgacattccactcaaagtcagcgctgtttgcctccttctctgtccacaga
aatatcgccgtctctttcgccgctgcgtccgctatctctttcgccaccgtttgtagcgttacctagcgtcaatgtccgc
cttcagttgcactttgtcagcggtttcgtgacgaagctccaagcggtttacgccatcaattaaacacaaagtgctgt
gccaaaactcctctcgcttcttatttttgtttgtttttgagtgattggggtggtgattggttttgggtgggtaagcagggg
aaagtgtgaaaaatcccggcaatgggccaagaggatcaggagctattaattcgcggaggcagcaaacaccc
atctgccgagcatctgaacaatgtgagtagtacatgtgcatacatcttaagttcacttgatctataggaactgcgatt
gcaacatcaaattgtctgcggcgtgagaactgcgacccacaaaaatcccaaaccgcaattgcacaaacaaat
agtgacacgaaacagattattctggtagctgttctcgctatataagacaatttttgagatcatatcatgatcaagac
atctaaaggcattcattttcgactatattctttttttacaaaaaatataacaaccagatattttaagctgatcctagatgc
acaaaaaataaataaaagtataaacctacttcgtaggatacttcggggtactttttgttcggggttagatgagcata
acgcttgtagttgatatttgagatcccctatcattgcagggtgacagcggagcggcttcgcagagctgcattaacc
agggcttcgggcaggccaaaaactacggcacgctccggccacccagtccgccggaggactccggttcaggg
agcggccaactagccgagaacctcacctatgcctggcacaatatggacatctttggggcggtcaatcagccgg
gctccggatggcggcagctggtcaaccggacacgcggactattctgcaacgagcgacacataccggcgccc
aggaaacatttgctcaagaacggtgagtttctattcgcagtcggctgatctgtgtgaaatcttaataaagggtcca
attaccaatttgaaactcagtttgcggcgtggcctatccgggcgaacttttggccgtgatgggcagttccggtgcc
ggaaagacgaccctgctgaatgcccttgcctttcgatcgccgcagggcatccaagtatcgccatccgggatgcg
actgctcaatggccaacctgtggacgccaaggagatgcaggccaggtgcgcctatgtccagcaggatgacct
ctttatcggctccctaacggccagggaacacctgattttccaagccatggtgcggatgccacgacatctgaccta
tcggcagcgagtggcccgcgtggatcaggtgatccaggagctttcgctcagcaaatgtcagcacacgatcatc

ggtgtgcccggcagggtgaaaggtctgtccggcggagaaaggaagcgtctggcattcgcctccgaggctctaa
ccgatccgccgcttctgatctgcgatgagcccacctccggactggactcctttaccgcccacagcgtcgtccagg
tgctgaagaagctgtcgcagaagggcaagaccgtcatcctgaccattcatcagccgtcttccgagctgtttgagc
tctttgacaagatccttctgatggccgagggcagggtagctttcttgggcactcccagcgaagccgtcgacttctttt
cctagtgagttcgatgtgtttattaagggtatctagtattacataacatctcaactcctatccagcgtgggtgcccagt
gtcctaccaactacaatccggcggacttttacgtacaggtgttggccgttgtgcccggacgggagatcgagtccc
gtgatcggatcgccaagatatgcgacaattttgccattagcaaagtagcccgggatatggagcagttgttggcca
ccaaaaatctggagaagccactggagcagccggagaatgggtacacctacaaggccacctggttcatgcagt
tccgggcggtcctgtggcgatcctggctgtcggtgctcaaggaaccactcctcgtaaaagtgcgacttattcaga
caacggtgagtggttccagtggaaacaaatgatataacgcttacaattcttggaaacaaattcgctagattttaga
tagaattgcctgattccacacccttcttagttttttcaatgagatgtatagtttatagttttgcagaagataaataaattt
catttaactcgcgaatattaatgagatgcgagtaacattttaatttgcagatggttgccatcttgattggcctcatctttt
tgggccaacaactcacgcaagtgggtgtgatgaatatcaacggagccatcttcctcttcctgaccaacatgacct
ttcaaaacgtctttgccacgataaatgtaagtcatgtttagaatacatttgcatttcaataatttactaactttctaatga
atcgattcgatttaggtgttcacctcagagctgccagtttttatgagggaggcccgaagtcgactttatcgctgtgac
acatactttctgggcaaaacgattgccgaattgccgctttttctcacagtgccactggtcttcacggcgattgcctat
ccgatgatcggactgcgggccggagtgctgcacttcttcaactgcctggcgctggtcactctggtggccaatgtgt
caacgtccttcggatatctaatatcctgcgccagctcctcgacctcgatggcgctgtctgtgggtccgccggttatc
ataccattcctgctctttggcggcttcttcttgaactcgggctcggtgccagtatacctcaaatggttgtcgtacctctc
atggttccgttacgccaacgagggtctgctgattaaccaatgggcggacgtggagccgggcgaaattagctgc
acatcgtcgaacaccacgtgccccagttcgggcaaggtcatcctggagacgcttaacttctccgccgccgatct
gccgctggactacgtgggtctggccattctcatcgtgagcttccgggtgctcgcatatctggctctaagacttcggg
cccgacgcaaggagtagccgacatatatccgaaataactgcttgttttttttttaccattattaccatcgtgtttactgt
ttattgccccctcaaaaagctaatgtaattatatttgtgccaataaaaacaagatatgacctatagaatacaagtat
ttccccttcgaacatccccacaagtagactttggatttgtcttctaaccaaaagacttacacacctgcataccttac
atcaaaaactcgtttatcgctacataaaacaccgggatatatttttatatacatacttttcaaatcgcgcgccctctt
cataattcacctccaccacaccacgtttcgtagttgctctttcgctgtctcccacccgctctccgcaacacattcacc
ttttgttcgacgaccttggagcgactgtcgttagttccgcgcgattcggttcgctcaaatggttccgagtggttcatttc
gtctcaatagaaattagtaataaatatttgtatgtacaatttatttgctccaatatatttgtatatatttccctcacagctat
atttattctaatttaatattatgactttttaaggtaatttttttgtgacctgttcggagtgattagcgttacaatttgaactgaa
agtgacatccagtgtttgttccttgtgtagatgcatctcaaaaaaaatggtgggcataatagtgttgtttatatatatca
aaaataacaactataataataagaatacatttaatttagaaaatgcttggatttcactggaactagaattaattcgg
gcggccgccaccgcggtggagctccaattcgccctatagtgagtcgtattacgcgcgctcactggccgtcgtttta
caacgtcgtgactgggaaaaccctggcgttacccaacttaatcgccttgcagcacatccccctttcgccagctgg
cgtaatagcgaagaggcccgcaccgatcgcccttcccaacagttgcgcagcctgaatggcgaatggacgcg
ccctgtagcggcgcattaagcgcggcgggtgtggtggttacgcgcagcgtgaccgctacacttgccagcgccct
agcgcccgctcctttcgctttcttcccttcctttctcgccacgttcgccggctttccccgtcaagctctaaatcggggg
ctccctttagggttccgatttagtgctttacggcacctcgaccccaaaaaacttgattagggtgatggttcacgtagt
gggccatcgccctgatagacggtttttcgccctttgacgttggagtccacgttctttaatagtggactcttgttccaaa
ctggaacaacactcaaccctatctcggtctattcttttgatttataagggattttgccgatttcggcctattggttaaaa
aatgagctgatttaacaaaaatttaacgcgaattttaacaaaatattaacgcttacaatttag

pB-ARE-Red

Plasmid backbone:pBluescript II KS(+)
attB: 2208-2516
gypsy insulator: 2532-2927; 4174-4559
4X ARE: 2937-3016
Ds.Red.T4: 3023-4144
mini-white: 5047-7919

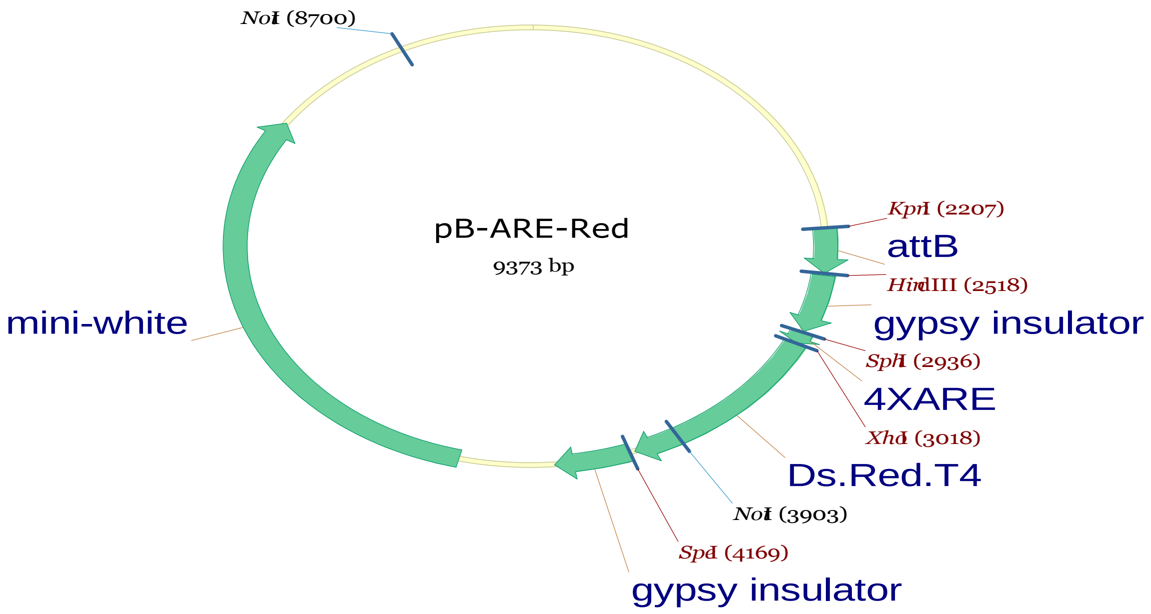

gtggcacttttcggggaaatgtgcgcggaacccctatttgtttatttttctaaatacattcaaatatgtatccgctcatg
agacaataaccctgataaatgcttcaataatattgaaaaaggaagagtatgagtattcaacatttccgtgtcgccc
ttattcccttttttgcggcattttgccttcctgtttttgctcacccagaaacgctggtgaaagtaaaagatgctgaagat
cagttgggtgcacgagtgggttacatcgaactggatctcaacagcggtaagatccttgagagttttcgccccgaa
gaacgttttccaatgatgagcacttttaaagttctgctatgtggcgcggtattatcccgtattgacgccgggcaaga
gcaactcggtcgccgcatacactattctcagaatgacttggttgagtactcaccagtcacagaaaagcatcttac
ggatggcatgacagtaagagaattatgcagtgctgccataaccatgagtgataacactgcggccaacttacttct
gacaacgatcggaggaccgaaggagctaaccgcttttttgcacaacatgggggatcatgtaactcgccttgatc
gttgggaaccggagctgaatgaagccataccaaacgacgagcgtgacaccacgatgcctgtagcaatggca
acaacgttgcgcaaactattaactggcgaactacttactctagcttcccggcaacaattaatagactggatggag
gcggataaagttgcaggaccacttctgcgctcggcccttccggctggctggtttattgctgataaatctggagccg
gtgagcgtgggtctcgcggtatcattgcagcactggggccagatggtaagccctcccgtatcgtagttatctacac
gacggggagtcaggcaactatggatgaacgaaatagacagatcgctgagataggtgcctcactgattaagcat
tggtaactgtcagaccaagtttactcatatatactttagattgatttaaaacttcatttttaatttaaaaggatctaggtg
aagatcctttttgataatctcatgaccaaaatcccttaacgtgagttttcgttccactgagcgtcagaccccgtaga
aaagatcaaaggatcttcttgagatcctttttttctgcgcgtaatctgctgcttgcaaacaaaaaaaccaccgctac
cagcggtggtttgtttgccggatcaagagctaccaactctttttccgaaggtaactggcttcagcagagcgcagat
accaaatactgtccttctagtgtagccgtagttaggccaccacttcaagaactctgtagcaccgcctacatacctc
gctctgctaatcctgttaccagtggctgctgccagtggcgataagtcgtgtcttaccgggttggactcaagacgat
agttaccggataaggcgcagcggtcgggctgaacggggggttcgtgcacacagcccagcttggagcgaacg
acctacaccgaactgagatacctacagcgtgagctatgagaaagcgccacgcttcccgaagggagaaaggc
ggacaggtatccggtaagcggcagggtcggaacaggagagcgcacgagggagcttccagggggaaacgc
ctggtatctttatagtcctgtcgggtttcgccacctctgacttgagcgtcgatttttgtgatgctcgtcaggggggcgg
agcctatggaaaaacgccagcaacgcggcctttttacggttcctggccttttgctggccttttgctcacatgttctttc
ctgcgttatcccctgattctgtggataaccgtattaccgcctttgagtgagctgataccgctcgccgcagccgaac
gaccgagcgcagcgagtcagtgagcgaggaagcggaagagcgcccaatacgcaaaccgcctctccccgc
gcgttggccgattcattaatgcagctggcacgacaggtttcccgactggaaagcgggcagtgagcgcaacgca
attaatgtgagttagctcactcattaggcaccccaggctttacactttatgcttccggctcgtatgttgtgtggaattgt
gagcggataacaatttcacacaggaaacagctatgaccatgattacgccaagcgcgcaattaaccctcactaa
agggaacaaaagctgggtaccgggccccccctcgacgatgtaggtcacggtctcgaagccgcggtgcgggt
gccagggcgtgcccttgggctccccgggcgcgtactccacctcacccatctggtccatcatgatgaacgggtcg
aggtggcggtagttgatcccggcgaacgcgcggcgcaccgggaagccctcgccctcgaaaccgctgggcgc
ggtggtcacggtgagcacgggacgtgcgacggcgtcggcgggtgcggatacgcggggcagcgtcagcgggt
tctcgacggtcacggcgggcatgtcgaggtcgacggtatcgataagcttgggctgcatcacgtaataagtgtgc
gttgaatttattcgcaaaaacattgcatattttcggcaaagtaaaattttgttgcataccttatcaaaaaataagtgct

gcatactttttagagaaaccaaataattttttattgcatacccgtttttaataaaatacattgcataccctcttttaataa
aaaatattgcatactttgacgaaacaaattttcgttgcatacccaataaaagattattatattgcatacccgtttttaat
aaaatacattgcataccctcttttaataaaaaatattgcatacgttgacgaaacaaattttcgttgcatacccaataa
aagattattatattgcataccttttcttgccataccatttagccgatcaattgtgctcggcaacagcatgcgatttctgc
aatgtcatcgtgatttctgcaatgtcatcgtgatttctgcaatgtcatcgtgatttctgcaatgtcatcgtctcgaggag
cgccggagtataaatagaggcgcttcgtctacggagcgacaattcaattcaaacaagcaaagtgaacacgtc
gctaagcgaaagctaagcaaataaacaagcgcagctgaacaagctaaacaatctgcagccaagctgatcct
ctagggtacgcgctagagtcgagaggcctgtttaaacgatccaccggtcgccaccatggcctcctccgaggac
gtcatcaaggagttcatgcgcttcaaggtgcgcatggagggctccgtgaacggccacgagttcgagatcgagg
gcgagggcgagggccgcccctacgagggcacccagaccgccaagctgaaggtgaccaagggcggcccc
ctgcccttcgcctgggacatcctgtcccccagttccagtacggctccaaggtgtacgtgaagcaccccgccga
catccccgactacaagaagctgtccttccccgagggcttcaagtgggagcgcgtgatgaacttcgaggacggc
ggcgtggtgaccgtgacccaggactcctccctgcaggacggctgcttcatctacaaggtgaagttcatcggcgt
gaacttcccctccgacggccccgtaatgcagaagaagactatgggctgggagccctccaccgagcgcctgta
cccccgcgacggcgtgctgaagggcgagatccacaaggccctgaagctgaaggacggcggccactacctg
gtggagttcaagtccatctacatggccaagaagcccgtgcagctgcccggctactactacgtggactccaagct
ggacatcacctcccacaacgaggactacaccatcgtggagcagtacgagcgcgccgagggccgccaccac
ctgttcctgtagcggccgcgactctagatcataatcagccataccacatttgtagaggttttacttgctttaaaaaac
ctcccacacctccccctgaacctgaaacataaaatgaatgcaattgttgttgttaacttgtttattgcagcttataatg
gttacaaataaagcaatagcatcacaaatttcacaaataaagcatttttttcactgcattctagttgtggtttgtccaa
actcatcaatgtatcttaaggcgtaaattgtaagcgttaatgactagtcacgtaataagtgtgcgttgaatttattcgc
aaaaacattgcatattttcggcaaagtaaaattttgttgcataccttatcaaaaaataagtgctgcatactttttagag
aaaccaaataattttttattgcatacccgtttttaataaaatacattgcataccctcttttaataaaaaatattgcatact
ttgacgaaacaaattttcgttgcatacccaataaaagattattatattgcatacccgtttttaataaaatacattgcat
accctcttttaataaaaaatattgcatacgttgacgaaacaaattttcgttgcatacccaataaaagattattatattg
cataccttttcttgccataccatttagccgatcaattctagtatgtatgtaagttaataaaaccctttttttggagaatgta
gatttaaaaaaacatattttttttttatttttactgcactggacatcattgaacttatctgatcagttttaaatttacttcgat
ccaagggtatttgaagtaccaggttctttcgattacctctcactcaaaatgacattccactcaaagtcagcgctgttt
gcctccttctctgtccacagaaatatcgccgtctctttcgccgctgcgtccgctatctctttcgccaccgtttgtagcgt
tacctagcgtcaatgtccgccttcagttgcactttgtcagcggtttcgtgacgaagctccaagcggtttacgccatc
aattaaacacaaagtgctgtgccaaaactcctctcgcttcttatttttgtttgtttttttgagtgattggggtggtgattggtt
ttgggtgggtaagcaggggaaagtgtgaaaaatcccggcaatgggccaagaggatcaggagctattaattcg
cggaggcagcaaacacccatctgccgagcatctgaacaatgtgagtagtacatgtgcatacatcttaagttcact
tgatctataggaactgcgattgcaacatcaaattgtctgcggcgtgagaactgcgacccacaaaaatcccaaac
cgcaattgcacaaacaaatagtgacacgaaacagattattctggtagctgttctcgctatataagacaattttttga
gatcatatcatgatcaagacatctaaaggcattcattttcgactatattctttttttacaaaaaatataacaaccagat
attttaagctgatcctagatgcacaaaaaataaataaaagtataaacctacttcgtaggatacttcggggtactttttt
gttcggggttagatgagcataacgcttgtagttgatatttgagatcccctatcattgcagggtgacagcggagcgg
cttcgcagagctgcattaaccagggcttcgggcaggccaaaaactacggcacgctccggccacccagtccgc
cggaggactccggttcagggagcggccaactagccgagaacctcacctatgcctggcacaatatggacatctt
tggggcggtcaatcagccgggctccggatggcggcagctggtcaaccggacacgcggactattctgcaacga
gcgacacataccggcgcccaggaaacatttgctcaagaacggtgagtttctattcgcagtcggctgatctgtgtg
aaatcttaataaagggtccaattaccaatttgaaactcagtttgcggcgtggcctatccgggcgaacttttggccgt
gatgggcagttccggtgccggaaagacgaccctgctgaatgcccttgcctttcgatcgccgcagggcatccaa
gtatcgccatccgggatgcgactgctcaatggccaacctgtggacgccaaggagatgcaggccaggtgcgcc
tatgtccagcaggatgacctctttatcggctccctaacggccagggaacacctgattttccaagccatggtgcgg
atgccacgacatctgacctatcggcagcgagtggcccgcgtggatcaggtgatccaggagctttcgctcagca
aatgtcagcacacgatcatcggtgtgcccggcagggtgaaaggtctgtccggcggagaaaggaagcgtctgg
cattcgcctccgaggctctaaccgatccgccgcttctgatctgcgatgagcccacctccggactggactcctttac
cgcccacagcgtcgtccaggtgctgaagaagctgtcgcagaagggcaagaccgtcatcctgaccattcatca
gccgtcttccgagctgtttgagctctttgacaagatccttctgatggccgagggcagggtagctttcttgggcactcc
cagcgaagccgtcgacttcttttcctagtgagttcgatgtgtttattaagggtatctagtattacataacatctcaactc
ctatccagcgtgggtgcccagtgtcctaccaactacaatccggcggacttttacgtacaggtgttggccgttgtgc
ccggacgggagatcgagtcccgtgatcggatcgccaagatatgcgacaattttgccattagcaaagtagcccg
ggatatggagcagttgttggccaccaaaaatctggagaagccactggagcagccggagaatgggtacaccta
caaggccacctggttcatgcagttccgggcggtcctgtggcgatcctggctgtcggtgctcaaggaaccactcct
cgtaaaagtgcgacttattcagacaacggtgagtggttccagtggaaacaaatgatataacgcttacaattcttg

gaaacaaattcgctagattttagatagaattgcctgattccacacccttcttagttttttcaatgagatgtatagtttat
agttttgcagaagataaataaatttcatttaactcgcgaatattaatgagatgcgagtaacattttaatttgcagatg
gttgccatcttgattggcctcatctttttgggccaacaactcacgcaagtgggtgtgatgaatatcaacggagccat
cttcctcttcctgaccaacatgacctttcaaaacgtctttgccacgataaatgtaagtcatgtttagaatacatttgca
tttcaataatttactaactttctaatgaatcgattcgatttaggtgttcacctcagagctgccagtttttatgagggagg
cccgaagtcgactttatcgctgtgacacatactttctgggcaaaacgattgccgaattgccgctttttctcacagtgc
cactggtcttcacggcgattgcctatccgatgatcggactgcgggccggagtgctgcacttcttcaactgcctggc
gctggtcactctggtggccaatgtgtcaacgtccttcggatatctaatatcctgcgccagctcctcgacctcgatgg
cgctgtctgtgggtccgccggttatcataccattcctgctctttggcggcttcttcttgaactcgggctcggtgccagt
atacctcaaatggttgtcgtacctctcatggttccgttacgccaacgagggtctgctgattaaccaatgggcggac
gtggagccgggcgaaattagctgcacatcgtcgaacaccacgtgccccagttcgggcaaggtcatcctggag
acgcttaacttctccgccgccgatctgccgctggactacgtgggtctggccattctcatcgtgagcttccgggtgct
cgcatatctggctctaagacttcgggcccgacgcaaggagtagccgacatatatccgaaataactgcttgtttttt
ttttaccattattaccatcgtgtttactgtttattgccccctcaaaaagctaatgtaattatatttgtgccaataaaaaca
agatatgacctatagaatacaagtatttccccttcgaacatccccacaagtagactttggatttgtcttctaaccaa
aagacttacacacctgcataccttacatcaaaaactcgtttatcgctacataaaacaccgggatatatttttatata
catacttttcaaatcgcgcgccctcttcataattcacctccaccacaccacgtttcgtagttgctctttcgctgtctccc
acccgctctccgcaacacattcacctttttgttcgacgaccttggagcgactgtcgttagttccgcgcgattcggttcg
ctcaaatggttccgagtggttcatttcgtctcaatagaaattagtaataaatatttgtatgtacaatttatttgctccaat
atatttgtatatatttccctcacagctatatttattctaatttaatattatgactttttaaggtaatttttgtgacctgttcgga
gtgattagcgttacaatttgaactgaaagtgacatccagtgtttgttccttgtgtagatgcatctcaaaaaaatggtg
ggcataatagtgttgtttatatatatcaaaaataacaactataataataagaatacatttaatttagaaaatgcttgg
atttcactggaactagaattaattcgggcggccgccaccgcggtggagctccaattcgccctatagtgagtcgtat
tacgcgcgctcactggccgtcgttttacaacgtcgtgactgggaaaaccctggcgttacccaacttaatcgccttg
cagcacatccccctttcgccagctggcgtaatagcgaagaggcccgcaccgatcgcccttcccaacagttgcg
cagcctgaatggcgaatgggacgcgccctgtagcggcgcattaagcgcggcgggtgtggtggttacgcgcag
cgtgaccgctacacttgccagcgccctagcgcccgctcctttcgctttcttcccttcctttctcgccacgttcgccgg
ctttccccgtcaagctctaaatcgggggctccctttagggttccgatttagtgctttacggcacctcgaccccaaaa
aacttgattagggtgatggttcacgtagtgggccatcgccctgatagacggtttttcgccctttgacgttggagtcca
cgttctttaatagtggactcttgttccaaactggaacaacactcaaccctatctcggtctattcttttgatttataaggg
attttgccgatttcggcctattggttaaaaaatgagctgatttaacaaaaatttaacgcgaattttaacaaaatattaa
cgcttacaatttag

pB-TRE-Green

Plasmid backbone:pBluescript II KS(+)
attB: 2208-2516
gypsy insulator: 2532-2927; 4222-4607
4X TRE: 2937-3016
eGFP: 3023-4192
mini-white: 5095-7967

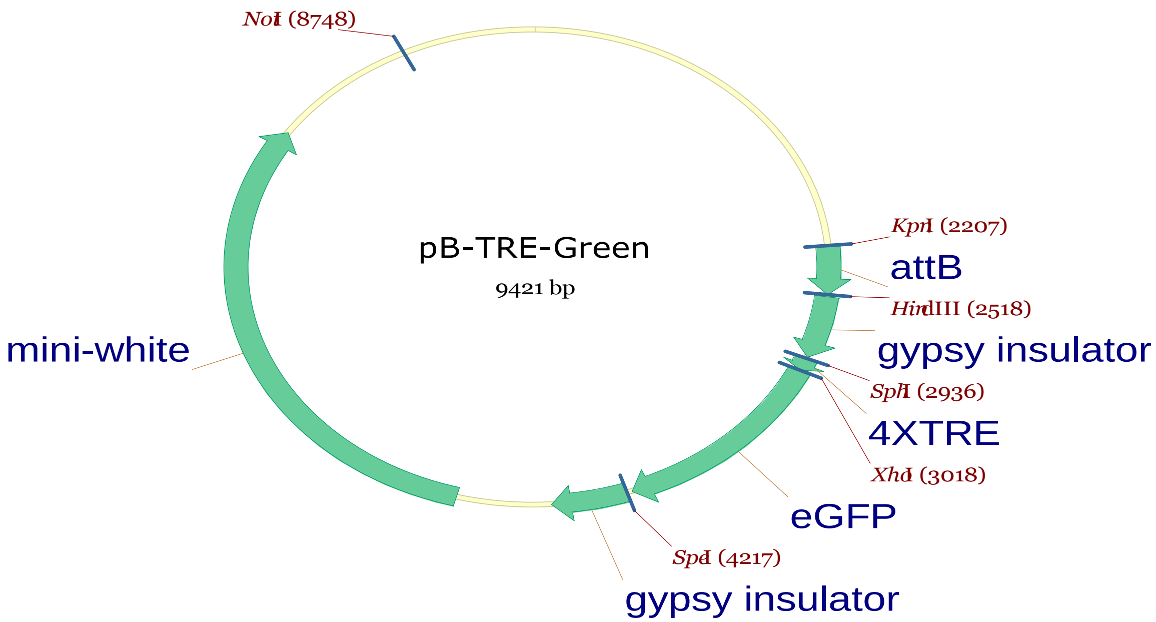

gtggcacttttcggggaaatgtgcgcggaacccctatttgtttatttttctaaatacattcaaatatgtatccgctcatg
agacaataaccctgataaatgcttcaataatattgaaaaaggaagagtatgagtattcaacatttccgtgtcgccc
ttattcccttttttgcggcattttgccttcctgtttttgctcacccagaaacgctggtgaaagtaaaagatgctgaagat
cagttgggtgcacgagtgggttacatcgaactggatctcaacagcggtaagatccttgagagttttcgccccgaa
gaacgttttccaatgatgagcacttttaaagttctgctatgtggcgcggtattatcccgtattgacgccgggcaaga
gcaactcggtcgccgcatacactattctcagaatgacttggttgagtactcaccagtcacagaaaagcatcttac
ggatggcatgacagtaagagaattatgcagtgctgccataaccatgagtgataacactgcggccaacttacttct
gacaacgatcggaggaccgaaggagctaaccgcttttttgcacaacatgggggatcatgtaactcgccttgatc
gttgggaaccggagctgaatgaagccataccaaacgacgagcgtgacaccacgatgcctgtagcaatggca
acaacgttgcgcaaactattaactggcgaactacttactctagcttcccggcaacaattaatagactggatggag
gcggataaagttgcaggaccacttctgcgctcggcccttccggctggctggtttattgctgataaatctggagccg
gtgagcgtgggtctcgcggtatcattgcagcactggggccagatggtaagccctcccgtatcgtagttatctacac
gacggggagtcaggcaactatggatgaacgaaatagacagatcgctgagataggtgcctcactgattaagcat
tggtaactgtcagaccaagtttactcatatatactttagattgatttaaaacttcatttttaatttaaaaggatctaggtg
aagatcctttttgataatctcatgaccaaaatcccttaacgtgagttttcgttccactgagcgtcagaccccgtaga
aaagatcaaaggatcttcttgagatcctttttttctgcgcgtaatctgctgcttgcaaacaaaaaaaccaccgctac
cagcggtggtttgtttgccggatcaagagctaccaactctttttccgaaggtaactggcttcagcagagcgcagat
accaaatactgtccttctagtgtagccgtagttaggccaccacttcaagaactctgtagcaccgcctacatacctc
gctctgctaatcctgttaccagtggctgctgccagtggcgataagtcgtgtcttaccgggttggactcaagacgat
agttaccggataaggcgcagcggtcgggctgaacggggggttcgtgcacacagcccagcttggagcgaacg
acctacaccgaactgagatacctacagcgtgagctatgagaaagcgccacgcttcccgaagggagaaaggc
ggacaggtatccggtaagcggcagggtcggaacaggagagcgcacgagggagcttccagggggaaacgc
ctggtatctttatagtcctgtcgggtttcgccacctctgacttgagcgtcgatttttgtgatgctcgtcaggggggcgg
agcctatggaaaaacgccagcaacgcggcctttttacggttcctggccttttgctggccttttgctcacatgttctttc
ctgcgttatcccctgattctgtggataaccgtattaccgcctttgagtgagctgataccgctcgccgcagccgaac
gaccgagcgcagcgagtcagtgagcgaggaagcggaagagcgcccaatacgcaaaccgcctctccccgc
gcgttggccgattcattaatgcagctggcacgacaggtttcccgactggaaagcgggcagtgagcgcaacgca
attaatgtgagttagctcactcattaggcaccccaggctttacactttatgcttccggctcgtatgttgtgtggaattgt
gagcggataacaatttcacacaggaaacagctatgaccatgattacgccaagcgcgcaattaaccctcactaa
agggaacaaaagctgggtaccgggccccccctcgacgatgtaggtcacggtctcgaagccgcggtgcgggt
gccagggcgtgcccttgggctccccgggcgcgtactccacctcacccatctggtccatcatgatgaacgggtcg
aggtggcggtagttgatcccggcgaacgcgcggcgcaccgggaagccctcgccctcgaaaccgctgggcgc
ggtggtcacggtgagcacgggacgtgcgacggcgtcggcgggtgcggatacgcggggcagcgtcagcgggt
tctcgacggtcacggcgggcatgtcgaggtcgacggtatcgataagcttgggctgcatcacgtaataagtgtgc
gttgaatttattcgcaaaaacattgcatattttcggcaaagtaaaattttgttgcataccttatcaaaaaataagtgct

gcatactttttagagaaaccaaataatttttattgcatacccgtttttaataaaatacattgcataccctcttttaataa
aaaatattgcatactttgacgaaacaaattttcgttgcatacccaataaaagattatatattgcatacccgtttttaat
aaaatacattgcataccctcttttaataaaaaatattgcatacgttgacgaaacaaattttcgttgcatacccaataa
aagattatatattgcataccttttcttgccataccatttagccgatcaattgtgctcggcaacagcatgcgatttcttat
gagtcatcgtgatttcttatgagtcatcgtgatttcttatgagtcatcgtgatttcttatgagtcatcgtctcgaggagcg
ccggagtataaatagaggcgcttcgtctacggagcgacaattcaattcaaacaagcaaagtgaacacgtcgct
aagcgaaagctaagcaaataaacaagcgcagctgaacaagctaaacaatctgcagccaagctgatcctcta
gggtacgcgctagagtcgagaggcctgtttaaacgatccaccggtcgccaccatggtgagcaagggcgagga
gctgttcaccggggtggtgcccatcctggtcgagctggacggcgacgtaaacggccacaagttcagcgtgtcc
ggcgagggcgagggcgatgccacctacggcaagctgaccctgaagttcatctgcaccaccggcaagctgcc
cgtgccctggcccaccctcgtgaccaccctgacctacggcgtgcagtgcttcagccgctaccccgaccacatg
aagcagcacgacttcttcaagtccgccatgcccgaaggctacgtccaggagcgcaccatcttcttcaaggacg
acggcaactacaagacccgcgccgaggtgaagttcgagggcgacaccctggtgaaccgcatcgagctgaa
gggcatcgacttcaaggaggacggcaacatcctggggcacaagctggagtacaactacaacagccacaac
gtctatatcatggccgacaagcagaagaacggcatcaaggtgaacttcaagatccgccacaacatcgaggac
ggcagcgtgcagctcgccgaccactaccagcagaacacccccatcggcgacggccccgtgctgctgcccga
caaccactacctgagcacccagtccgccctgagcaaagaccccaacgagaagcgcgatcacatggtcctgc
tggagttcgtgaccgccgccgggatcactctcggcatggacgagctgtacaagtaaagcggccggccgcgac
tctagatcataatcagccataccacatttgtagaggttttacttgctttaaaaaacctcccacacctccccctgaac
ctgaaacataaaatgaatgcaattgttgttgttaacttgtttattgcagcttataatggttacaaataaagcaatagc
atcacaaatttcacaaataaagcatttttttcactgcattctagttgtggtttgtccaaactcatcaatgtatcttaagg
cgtaaattgtaagcgttaatgactagtcacgtaataagtgtgcgttgaatttattcgcaaaaacattgcatattttcgg
caaagtaaaattttgttgcataccttatcaaaaaataagtgctgcatactttttagagaaaccaaataatttttattgc
atacccgtttttaataaaatacattgcataccctcttttaataaaaaatattgcatactttgacgaaacaaattttcgtt
gcatacccaataaaagattatatattgcatacccgtttttaataaaatacattgcataccctcttttaataaaaaata
ttgcatacgttgacgaaacaaattttcgttgcatacccaataaaagattatatattgcataccttttcttgccatacca
tttagccgatcaattctagtatgtatgtaagttaataaaacccttttttggagaatgtagatttaaaaaaacatattttttt
tttatttttactgcactggacatcattgaacttatctgatcagttttaaatttacttcgatccaagggtatttgaagtacc
aggttctttcgattacctctcactcaaaatgacattccactcaaagtcagcgctgtttgcctccttctctgtccacaga
aatatcgccgtctctttcgccgctgcgtccgctatctctttcgccaccgtttgtagcgttacctagcgtcaatgtccgc
cttcagttgcactttgtcagcggtttcgtgacgaagctccaagcggtttacgccatcaattaaacacaaagtgctgt
gccaaaactcctctcgcttcttatttttgtttgtttttttgagtgattggggtggtgattggttttgggtgggtaagcagggg
aaagtgtgaaaaatcccggcaatgggccaagaggatcaggagctattaattcgcggaggcagcaaacaccc
atctgccgagcatctgaacaatgtgagtagtacatgtgcatacatcttaagttcacttgatctataggaactgcgatt
gcaacatcaaattgtctgcggcgtgagaactgcgacccacaaaaatcccaaaccgcaattgcacaaacaaat
agtgacacgaaacagattattctggtagctgttctcgctatataagacaatttttgagatcatatcatgatcaagac
atctaaaggcattcattttcgactatattctttttttacaaaaaatataacaaccagatattttaagctgatcctagatgc
acaaaaaataaataaaagtataaacctacttcgtaggatacttcggggtactttttgttcggggttagatgagcata
acgcttgtagttgatatttgagatcccctatcattgcagggtgacagcggagcggcttcgcagagctgcattaacc
agggcttcgggcaggccaaaaactacggcacgctccggccacccagtccgccggaggactccggttcaggg
agcggccaactagccgagaacctcacctatgcctggcacaatatggacatctttggggcggtcaatcagccgg
gctccggatggcggcagctggtcaaccggacacgcggactattctgcaacgagcgacacataccggcgccc
aggaaacatttgctcaagaacggtgagtttctattcgcagtcggctgatctgtgtgaaatcttaataaagggtcca
attaccaatttgaaactcagtttgcggcgtggcctatccgggcgaacttttggccgtgatgggcagttccggtgcc
ggaaagacgaccctgctgaatgcccttgcctttcgatcgccgcagggcatccaagtatcgccatccgggatgcg
actgctcaatggccaacctgtggacgccaaggagatgcaggccaggtgcgcctatgtccagcaggatgacct
ctttatcggctccctaacggccagggaacacctgattttccaagccatggtgcggatgccacgacatctgaccta
tcggcagcgagtggcccgcgtggatcaggtgatccaggagctttcgctcagcaaatgtcagcacacgatcatc
ggtgtgcccggcagggtgaaaggtctgtccggcggagaaaggaagcgtctggcattcgcctccgaggctctaa
ccgatccgccgcttctgatctgcgatgagcccacctccggactggactcctttaccgcccacagcgtcgtccagg
tgctgaagaagctgtcgcagaagggcaagaccgtcatcctgaccattcatcagccgtcttccgagctgtttgagc
tctttgacaagatccttctgatggccgagggcagggtagctttcttgggcactcccagcgaagccgtcgacttctttt
cctagtgagttcgatgtgtttattaagggtatctagtattacataacatctcaactcctatccagcgtgggtgcccagt
gtcctaccaactacaatccggcggacttttacgtacaggtgttggccgttgtgcccggacgggagatcgagtccc
gtgatcggatcgccaagatatgcgacaattttgccattagcaaagtagcccgggatatggagcagttgttggcca
ccaaaaatctggagaagccactggagcagccggagaatgggtacacctacaaggccacctggttcatgcagt
tccgggcggtcctgtggcgatcctggctgtcggtgctcaaggaaccactcctcgtaaaagtgcgacttattcaga

caacggtgagtggttccagtggaaacaaatgatataacgcttacaattcttggaaacaaattcgctagattttaga
tagaattgcctgattccacacccttcttagttttttcaatgagatgtatagtttatagttttgcagaagataaataaattt
catttaactcgcgaatattaatgagatgcgagtaacattttaatttgcagatggttgccatcttgattggcctcatctttt
tgggccaacaactcacgcaagtgggtgtgatgaatatcaacggagccatcttcctcttcctgaccaacatgacct
ttcaaaacgtctttgccacgataaatgtaagtcatgtttagaatacatttgcatttcaataatttactaactttctaatga
atcgattcgatttaggtgttcacctcagagctgccagtttttatgagggaggcccgaagtcgactttatcgctgtgac
acatactttctgggcaaaacgattgccgaattgccgctttttctcacagtgccactggtcttcacggcgattgcctat
ccgatgatcggactgcgggccggagtgctgcacttcttcaactgcctggcgctggtcactctggtggccaatgtgt
caacgtccttcggatatctaatatcctgcgccagctcctcgacctcgatggcgctgtctgtgggtccgccggttatc
ataccattcctgctctttggcggcttcttcttgaactcgggctcggtgccagtatacctcaaatggttgtcgtacctctc
atggttccgttacgccaacgagggtctgctgattaaccaatgggcggacgtggagccgggcgaaattagctgc
acatcgtcgaacaccacgtgccccagttcgggcaaggtcatcctggagacgcttaacttctccgccgccgatct
gccgctggactacgtgggtctggccattctcatcgtgagcttccgggtgctcgcatatctggctctaagacttcggg
cccgacgcaaggagtagccgacatatatccgaaataactgcttgtttttttttttaccattattaccatcgtgtttactgt
ttattgccccctcaaaaagctaatgtaattatatttgtgccaataaaaacaagatatgacctatagaatacaagtat
ttccccttcgaacatccccacaagtagactttggatttgtcttctaaccaaaagacttacacacctgcataccttac
atcaaaaactcgtttatcgctacataaaacaccgggatatatttttatatacatacttttcaaatcgcgcgccctctt
cataattcacctccaccacaccacgtttcgtagttgctctttcgctgtctcccacccgctctccgcaacacattcacc
ttttgttcgacgaccttggagcgactgtcgttagttccgcgcgattcggttcgctcaaatggttccgagtggttcatttc
gtctcaatagaaattagtaataaatatttgtatgtacaatttatttgctccaatatatttgtatatatttccctcacagctat
atttattctaatttaatattatgactttttaaggtaatttttgtgacctgttcggagtgattagcgttacaatttgaactgaa
agtgacatccagtgtttgttccttgtgtagatgcatctcaaaaaaatggtgggcataatagtgttgtttatatatatca
aaaataacaactataataataagaatacatttaatttagaaaatgcttggatttcactggaactagaattaattcgg
gcggccgccaccgcggtggagctccaattcgccctatagtgagtcgtattacgcgcgctcactggccgtcgtttta
caacgtcgtgactgggaaaaccctggcgttacccaacttaatcgccttgcagcacatccccctttcgccagctgg
cgtaatagcgaagaggcccgcaccgatcgcccttcccaacagttgcgcagcctgaatggcgaatggacgcg
ccctgtagcggcgcattaagcgcggcgggtgtggtggttacgcgcagcgtgaccgctacacttgccagcgccct
agcgcccgctcctttcgctttcttcccttcctttctcgccacgttcgccggctttccccgtcaagctctaaatcgggg
ctccctttagggttccgatttagtgctttacggcacctcgaccccaaaaaacttgattagggtgatggttcacgtagt
gggccatcgccctgatagacggtttttcgccctttgacgttggagtccacgttctttaatagtggactcttgttccaaa
ctggaacaacactcaaccctatctcggtctattcttttgatttataagggattttgccgatttcggcctattggttaaaa
aatgagctgatttaacaaaaatttaacgcgaattttaacaaaatattaacgcttacaatttag

pB-TRE-Red

Plasmid backbone:pBluescript II KS(+)
attB: 2208-2516
gypsy insulator: 2532-2927; 4174-4559
4X TRE: 2937-3016
Ds.Red.T4: 3023-4144
mini-white: 5047-7919

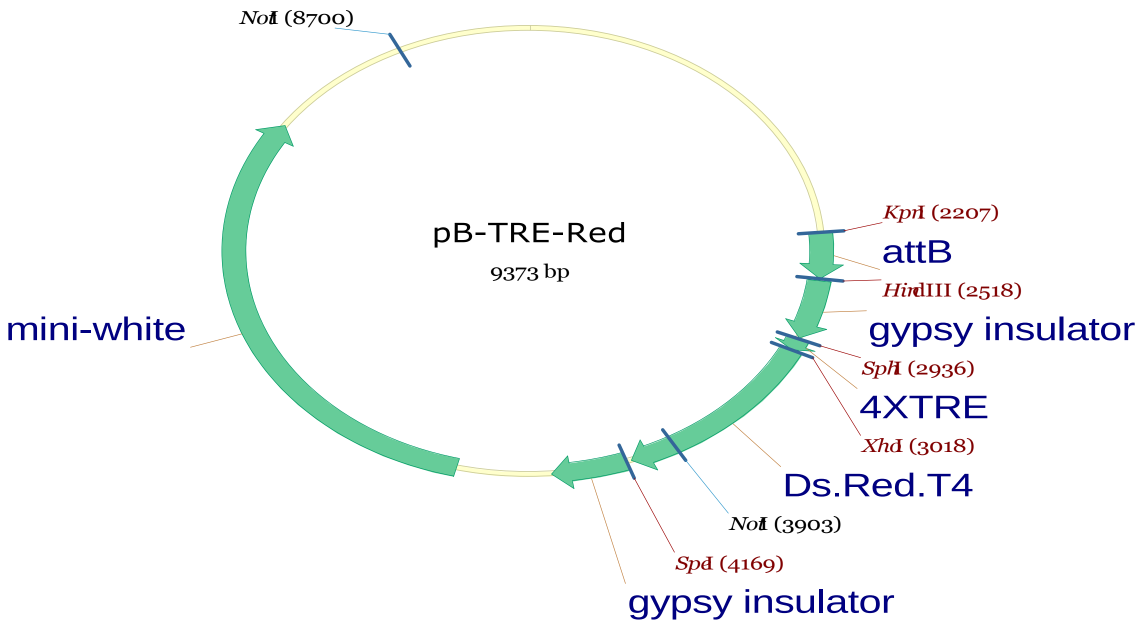

gtggcacttttcggggaaatgtgcgcggaacccctatttgtttatttttctaaatacattcaaatatgtatccgctcatg
agacaataaccctgataaatgcttcaataatattgaaaaaggaagagtatgagtattcaacatttccgtgtcgccc
ttattcccttttttgcggcattttgccttcctgtttttgctcacccagaaacgctggtgaaagtaaaagatgctgaagat
cagttgggtgcacgagtgggttacatcgaactggatctcaacagcggtaagatccttgagagttttcgccccgaa
gaacgttttccaatgatgagcacttttaaagttctgctatgtggcgcggtattatcccgtattgacgccgggcaaga
gcaactcggtcgccgcatacactattctcagaatgacttggttgagtactcaccagtcacagaaaagcatcttac
ggatggcatgacagtaagagaattatgcagtgctgccataaccatgagtgataacactgcggccaacttacttct
gacaacgatcggaggaccgaaggagctaaccgcttttttgcacaacatgggggatcatgtaactcgccttgatc
gttgggaaccggagctgaatgaagccataccaaacgacgagcgtgacaccacgatgcctgtagcaatggca
acaacgttgcgcaaactattaactggcgaactacttactctagcttcccggcaacaattaatagactggatggag
gcggataaagttgcaggaccacttctgcgctcggcccttccggctggctggtttattgctgataaatctggagccg
gtgagcgtgggtctcgcggtatcattgcagcactggggccagatggtaagccctcccgtatcgtagttatctacac
gacggggagtcaggcaactatggatgaacgaaatagacagatcgctgagataggtgcctcactgattaagcat
tggtaactgtcagaccaagtttactcatatatactttagattgatttaaaacttcatttttaatttaaaaggatctaggtg
aagatcctttttgataatctcatgaccaaaatcccttaacgtgagttttcgttccactgagcgtcagaccccgtaga
aaagatcaaaggatcttcttgagatcctttttttctgcgcgtaatctgctgcttgcaaacaaaaaaaccaccgctac
cagcggtggtttgtttgccggatcaagagctaccaactctttttccgaaggtaactggcttcagcagagcgcagat
accaaatactgtccttctagtgtagccgtagttaggccaccacttcaagaactctgtagcaccgcctacatacctc
gctctgctaatcctgttaccagtggctgctgccagtggcgataagtcgtgtcttaccgggttggactcaagacgat
agttaccggataaggcgcagcggtcgggctgaacggggggttcgtgcacacagcccagcttggagcgaacg
acctacaccgaactgagatacctacagcgtgagctatgagaaagcgccacgcttcccgaagggagaaaggc
ggacaggtatccggtaagcggcagggtcggaacaggagagcgcacgagggagcttccagggggaaacgc
ctggtatctttatagtcctgtcgggtttcgccacctctgacttgagcgtcgatttttgtgatgctcgtcaggggggcgg
agcctatggaaaaacgccagcaacgcggcctttttacggttcctggccttttgctggccttttgctcacatgttctttc
ctgcgttatcccctgattctgtggataaccgtattaccgcctttgagtgagctgataccgctcgccgcagccgaac
gaccgagcgcagcgagtcagtgagcgaggaagcggaagagcgcccaatacgcaaaccgcctctccccgc
gcgttggccgattcattaatgcagctggcacgacaggtttcccgactggaaagcgggcagtgagcgcaacgca
attaatgtgagttagctcactcattaggcaccccaggctttacactttatgcttccggctcgtatgttgtgtggaattgt
gagcggataacaatttcacacaggaaacagctatgaccatgattacgccaagcgcgcaattaaccctcactaa
agggaacaaaagctgggtaccgggccccccctcgacgatgtaggtcacggtctcgaagccgcggtgcgggt
gccagggcgtgcccttgggctccccgggcgcgtactccacctcacccatctggtccatcatgatgaacgggtcg
aggtggcggtagttgatcccggcgaacgcgcggcgcaccgggaagccctcgccctcgaaaccgctgggcgc
ggtggtcacggtgagcacgggacgtgcgacggcgtcggcgggtgcggatacgcggggcagcgtcagcgggt
tctcgacggtcacggcgggcatgtcgaggtcgacggtatcgataagcttgggctgcatcacgtaataagtgtgc

gttgaatttattcgcaaaaacattgcatattttcggcaaagtaaaattttgttgcataccttatcaaaaaataagtgct
gcatactttttagagaaaccaaataatttttttattgcatacccgtttttaataaaatacattgcataccctcttttaataa
aaaatattgcatactttgacgaaacaaattttcgttgcatacccaataaaagattattatattgcatacccgtttttaat
aaaatacattgcataccctcttttaataaaaaaatattgcatacgttgacgaaacaaattttcgttgcatacccaataa
aagattattatattgcatacctttttcttgccataccatttagccgatcaattgtgctcggcaacagcatgcgatttcttat
gagtcatcgtgatttcttatgagtcatcgtgatttcttatgagtcatcgtgatttcttatgagtcatcgtctcgaggagcg
ccggagtataaatagaggcgcttcgtctacggagcgacaattcaattcaaacaagcaaagtgaacacgtcgct
aagcgaaagctaagcaaataaacaagcgcagctgaacaagctaaacaatctgcagccaagctgatcctcta
gggtacgcgctagagtcgagaggcctgtttaaacgatccaccggtcgccaccatggcctcctccgaggacgtc
atcaaggagttcatgcgcttcaaggtgcgcatggagggctccgtgaacggccacgagttcgagatcgagggc
gagggcgagggccgcccctacgagggcacccagaccgccaagctgaaggtgaccaagggcggccccctg
cccttcgcctgggacatcctgtcccccagttccagtacggctccaaggtgtacgtgaagcaccccgccgacat
ccccgactacaagaagctgtccttccccgagggcttcaagtgggagcgcgtgatgaacttcgaggacggcgg
cgtggtgaccgtgacccaggactcctccctgcaggacggctgcttcatctacaaggtgaagttcatcggcgtga
acttcccctccgacggccccgtaatgcagaagaagactatgggctgggagccctccaccgagcgcctgtacc
cccgcgacggcgtgctgaagggcgagatccacaaggccctgaagctgaaggacggcggccactacctggt
ggagttcaagtccatctacatggccaagaagcccgtgcagctgcccggctactactacgtggactccaagctg
gacatcacctcccacaacgaggactacaccatcgtggagcagtacgagcgcgccgagggccgccaccacct
gttcctgtagcggccgcgactctagatcataatcagccataccacatttgtagaggttttacttgctttaaaaaacct
cccacacctccccctgaacctgaaacataaaatgaatgcaattgttgttgttaacttgtttattgcagcttataatggt
tacaaataaagcaatagcatcacaaatttcacaaataaagcatttttttcactgcattctagttgtggtttgtccaaa
ctcatcaatgtatcttaaggcgtaaattgtaagcgttaatgactagtcacgtaataagtgtgcgttgaatttattcgca
aaaacattgcatattttcggcaaagtaaaattttgttgcataccttatcaaaaaataagtgctgcatactttttagaga
aaccaaataatttttttattgcatacccgtttttaataaaatacattgcataccctcttttaataaaaaaatattgcatacttt
gacgaaacaaattttcgttgcatacccaataaaagattattatattgcatacccgtttttaataaaatacattgcata
ccctcttttaataaaaaaatattgcatacgttgacgaaacaaattttcgttgcatacccaataaaagattattatattgc
atacctttttcttgccataccatttagccgatcaattctagtatgtatgtaagttaataaaaccctttttttggagaatgtag
atttaaaaaaacatattttttttttatttttttactgcactggacatcattgaacttatctgatcagttttaaatttacttcgatc
caagggtatttgaagtaccaggttctttcgattacctctcactcaaaatgacattccactcaaagtcagcgctgtttg
cctccttctctgtccacagaaatatcgccgtctctttcgccgctgcgtccgctatctctttcgccaccgtttgtagcgtt
acctagcgtcaatgtccgccttcagttgcactttgtcagcggtttcgtgacgaagctccaagcggtttacgccatca
attaaacacaaagtgctgtgccaaaactcctctcgcttcttatttttgtttgtttttttgagtgattggggtggtgattggttt
tgggtgggtaagcaggggaaagtgtgaaaaatcccggcaatgggccaagaggatcaggagctattaattcgc
ggaggcagcaaacacccatctgccgagcatctgaacaatgtgagtagtacatgtgcatacatcttaagttcactt
gatctataggaactgcgattgcaacatcaaattgtctgcggcgtgagaactgcgacccacaaaaatcccaaac
cgcaattgcacaaacaaatagtgacacgaaacagattattctggtagctgttctcgctatataagacaatttttga
gatcatatcatgatcaagacatctaaaggcattcattttcgactatattctttttttacaaaaaatataacaaccagat
attttaagctgatcctagatgcacaaaaaataaataaaagtataaacctacttcgtaggatacttcggggtacttttt
gttcggggttagatgagcataacgcttgtagttgatatttgagatcccctatcattgcagggtgacagcggagcgg
cttcgcagagctgcattaaccagggcttcgggcaggccaaaaactacggcacgctccggccacccagtccgc
cggaggactccggttcagggagcggccaactagccgagaacctcacctatgcctggcacaatatggacatctt
tggggcggtcaatcagccgggctccggatggcggcagctggtcaaccggacacgcggactattctgcaacga
gcgacacataccggcgcccaggaaacatttgctcaagaacggtgagtttctattcgcagtcggctgatctgtgtg
aaatcttaataaagggtccaattaccaatttgaaactcagtttgcggcgtggcctatccgggcgaacttttggccgt
gatgggcagttccggtgccggaaagacgaccctgctgaatgcccttgcctttcgatcgccgcagggcatccaa
gtatcgccatccgggatgcgactgctcaatggccaacctgtggacgccaaggagatgcaggccaggtgcgcc
tatgtccagcaggatgacctctttatcggctccctaacggccagggaacacctgattttccaagccatggtgcgg
atgccacgacatctgacctatcggcagcgagtggcccgcgtggatcaggtgatccaggagctttcgctcagca
aatgtcagcacacgatcatcggtgtgcccggcagggtgaaaggtctgtccggcggagaaaggaagcgtctgg
cattcgcctccgaggctctaaccgatccgccgcttctgatctgcgatgagcccacctccggactggactcctttac
cgcccacagcgtcgtccaggtgctgaagaagctgtcgcagaagggcaagaccgtcatcctgaccattcatca
gccgtcttccgagctgtttgagctctttgacaagatccttctgatggccgagggcagggtagctttcttgggcactcc
cagcgaagccgtcgacttcttttcctagtgagttcgatgtgtttattaagggtatctagtattacataacatctcaactc
ctatccagcgtgggtgcccagtgtcctaccaactacaatccggcggacttttacgtacaggtgttggccgttgtgc
ccggacgggagatcgagtcccgtgatcggatcgccaagatatgcgacaattttgccattagcaaagtagcccg
ggatatggagcagttgttggccaccaaaaatctggagaagccactggagcagccggagaatgggtacaccta
caaggccacctggttcatgcagttccgggcggtcctgtggcgatcctggctgtcggtgctcaaggaaccactcct

cgtaaaagtgcgacttattcagacaacggtgagtggttccagtggaaacaaatgatataacgcttacaattcttg
gaaacaaattcgctagattttagatagaattgcctgattccacacccttcttagtttttttcaatgagatgtatagtttat
agttttgcagaagataaataaatttcatttaactcgcgaatattaatgagatgcgagtaacattttaatttgcagatg
gttgccatcttgattggcctcatctttttgggccaacaactcacgcaagtgggtgtgatgaatatcaacggagccat
cttcctcttcctgaccaacatgacctttcaaaacgtctttgccacgataaatgtaagtcatgtttagaatacatttgca
tttcaataatttactaactttctaatgaatcgattcgatttaggtgttcacctcagagctgccagtttttatgagggagg
cccgaagtcgactttatcgctgtgacacatactttctgggcaaaacgattgccgaattgccgctttttctcacagtgc
cactggtcttcacggcgattgcctatccgatgatcggactgcgggccggagtgctgcacttcttcaactgcctggc
gctggtcactctggtggccaatgtgtcaacgtccttcggatatctaatatcctgcgccagctcctcgacctcgatgg
cgctgtctgtgggtccgccggttatcataccattcctgctctttggcggcttcttcttgaactcgggctcggtgccagt
atacctcaaatggttgtcgtacctctcatggttccgttacgccaacgagggtctgctgattaaccaatgggcggac
gtggagccgggcgaaattagctgcacatcgtcgaacaccacgtgccccagttcgggcaaggtcatcctggag
acgcttaacttctccgccgccgatctgccgctggactacgtgggtctggccattctcatcgtgagcttccgggtgct
cgcatatctggctctaagacttcgggcccgacgcaaggagtagccgacatatatccgaaataactgcttgtttttt
ttttaccattattaccatcgtgtttactgtttattgccccctcaaaaagctaatgtaattatatttgtgccaataaaaaca
agatatgacctatagaatacaagtatttccccttcgaacatccccacaagtagactttggatttgtcttctaaccaa
aagacttacacacctgcataccttacatcaaaaactcgtttatcgctacataaaacaccgggatatatttttatata
catacttttcaaatcgcgcgccctcttcataattcacctccaccacaccacgtttcgtagttgctctttcgctgtctccc
acccgctctccgcaacacattcaccttttgttcgacgaccttggagcgactgtcgttagttccgcgcgattcggttcg
ctcaaatggttccgagtggttcatttcgtctcaatagaaattagtaataaatatttgtatgtacaatttatttgctccaat
atatttgtatatatttccctcacagctatatttattctaatttaatattatgactttttaaggtaatttttttgtgacctgttcgga
gtgattagcgttacaatttgaactgaaagtgacatccagtgtttgttccttgtgtagatgcatctcaaaaaaatggtg
ggcataatagtgttgtttatatatatcaaaaataacaactataataataagaatacatttaatttagaaaatgcttgg
atttcactggaactagaattaattcgggcggccgccaccgcggtggagctccaattcgccctatagtgagtcgtat
tacgcgcgctcactggccgtcgttttacaacgtcgtgactgggaaaaccctggcgttacccaacttaatcgccttg
cagcacatccccctttcgccagctggcgtaatagcgaagaggcccgcaccgatcgcccttcccaacagttgcg
cagcctgaatggcgaatgggacgcgccctgtagcggcgcattaagcgcggcgggtgtggtggttacgcgcag
cgtgaccgctacacttgccagcgccctagcgcccgctcctttcgctttcttcccttcctttctcgccacgttcgccgg
ctttccccgtcaagctctaaatcgggggctccctttagggttccgatttagtgctttacggcacctcgaccccaaaa
aacttgattagggtgatggttcacgtagtgggccatcgccctgatagacggtttttcgccctttgacgttggagtcca
cgttctttaatagtggactcttgttccaaactggaacaacactcaaccctatctcggtctattcttttgatttataaggg
attttgccgatttcggcctattggttaaaaaatgagctgatttaacaaaaatttaacgcgaattttaacaaaatattaa
cgcttacaatttag

pB-mRE-Red

Plasmid backbone:pBluescript II KS(+)
attB: 2208-2516
gypsy insulator: 2532-2927; 4174-4559
4X mRE: 2937-3016
Ds.Red.T4: 3023-4144
mini-white: 5047-7919

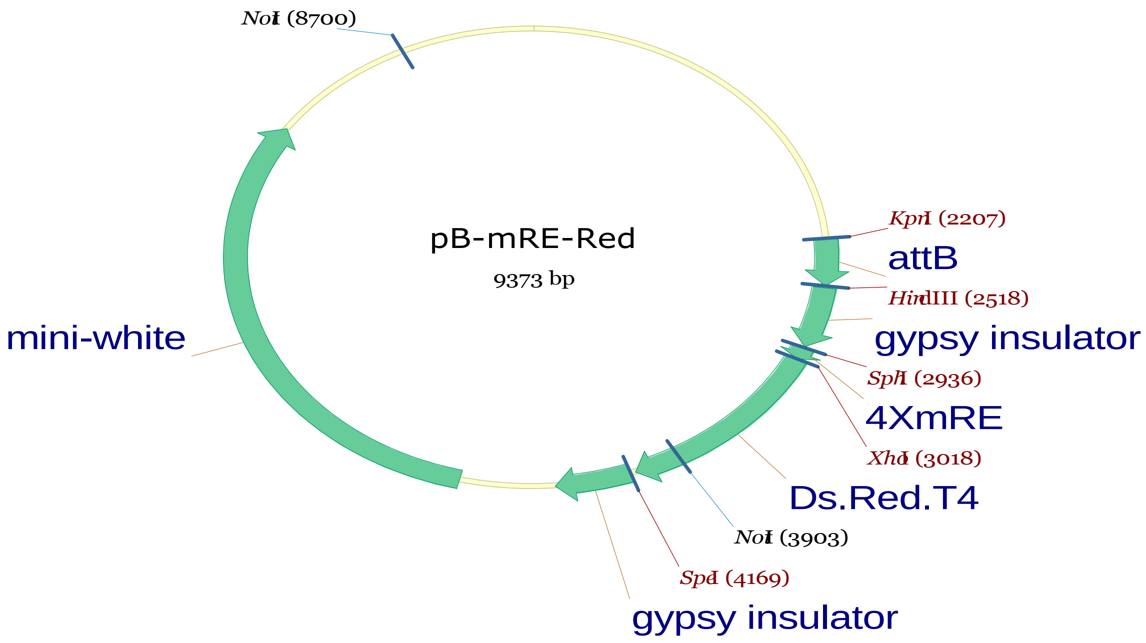

gtggcacttttcggggaaatgtgcgcggaacccctatttgtttatttttctaaatacattcaaatatgtatccgctcatg
agacaataaccctgataaatgcttcaataatattgaaaaaggaagagtatgagtattcaacatttccgtgtcgccc
ttattcccttttttgcggcattttgccttcctgtttttgctcacccagaaacgctggtgaaagtaaaagatgctgaagat
cagttgggtgcacgagtgggttacatcgaactggatctcaacagcggtaagatccttgagagttttcgccccgaa
gaacgttttccaatgatgagcacttttaaagttctgctatgtggcgcggtattatcccgtattgacgccgggcaaga
gcaactcggtcgccgcatacactattctcagaatgacttggttgagtactcaccagtcacagaaaagcatcttac
ggatggcatgacagtaagagaattatgcagtgctgccataaccatgagtgataacactgcggccaacttacttct
gacaacgatcggaggaccgaaggagctaaccgcttttttgcacaacatgggggatcatgtaactcgccttgatc
gttgggaaccggagctgaatgaagccataccaaacgacgagcgtgacaccacgatgcctgtagcaatggca
acaacgttgcgcaaactattaactggcgaactacttactctagcttcccggcaacaattaatagactggatggag
gcggataaagttgcaggaccacttctgcgctcggcccttccggctggctggtttattgctgataaatctggagccg
gtgagcgtgggtctcgcggtatcattgcagcactggggccagatggtaagccctcccgtatcgtagttatctacac
gacggggagtcaggcaactatggatgaacgaaatagacagatcgctgagataggtgcctcactgattaagcat
tggtaactgtcagaccaagtttactcatatatactttagattgatttaaaacttcatttttaatttaaaaggatctaggtg
aagatcctttttgataatctcatgaccaaaatcccttaacgtgagttttcgttccactgagcgtcagaccccgtaga
aaagatcaaaggatcttcttgagatcctttttttctgcgcgtaatctgctgcttgcaaacaaaaaaaccaccgctac
cagcggtggtttgtttgccggatcaagagctaccaactctttttccgaaggtaactggcttcagcagagcgcagat
accaaatactgtccttctagtgtagccgtagttaggccaccacttcaagaactctgtagcaccgcctacatacctc
gctctgctaatcctgttaccagtggctgctgccagtggcgataagtcgtgtcttaccgggttggactcaagacgat
agttaccggataaggcgcagcggtcgggctgaacggggggttcgtgcacacagcccagcttggagcgaacg
acctacaccgaactgagatacctacagcgtgagctatgagaaagcgccacgcttcccgaagggagaaaggc
ggacaggtatccggtaagcggcagggtcggaacaggagagcgcacgagggagcttccagggggaaacgc
ctggtatctttatagtcctgtcgggtttcgccacctctgacttgagcgtcgatttttgtgatgctcgtcaggggggcgg
agcctatggaaaaacgccagcaacgcggcctttttacggttcctggccttttgctggccttttgctcacatgttctttc
ctgcgttatcccctgattctgtggataaccgtattaccgcctttgagtgagctgataccgctcgccgcagccgaac
gaccgagcgcagcgagtcagtgagcgaggaagcggaagagcgcccaatacgcaaaccgcctctccccgc
gcgttggccgattcattaatgcagctggcacgacaggtttcccgactggaaagcgggcagtgagcgcaacgca
attaatgtgagttagctcactcattaggcaccccaggctttacactttatgcttccggctcgtatgttgtgtggaattgt
gagcggataacaatttcacacaggaaacagctatgaccatgattacgccaagcgcgcaattaaccctcactaa
agggaacaaaagctgggtaccgggccccccctcgacgatgtaggtcacggtctcgaagccgcggtgcgggt
gccagggcgtgcccttgggctccccgggcgcgtactccacctcacccatctggtccatcatgatgaacgggtcg
aggtggcggtagttgatcccggcgaacgcgcggcgcaccgggaagccctcgccctcgaaaccgctgggcgc
ggtggtcacggtgagcacgggacgtgcgacggcgtcggcgggtgcggatacgcggggcagcgtcagcgggt
tctcgacggtcacggcgggcatgtcgaggtcgacggtatcgataagcttgggctgcatcacgtaataagtgtgc

gttgaatttattcgcaaaaacattgcatattttcggcaaagtaaaattttgttgcataccttatcaaaaaataagtgct
gcatactttttagagaaaccaaataatttttattgcatacccgtttttaataaaatacattgcataccctcttttaataa
aaaatattgcatactttgacgaaacaaattttcgttgcatacccaataaaagattattatattgcatacccgtttttaat
aaaatacattgcataccctcttttaataaaaaaatattgcatacgttgacgaaacaaattttcgttgcatacccaataa
aagattattatattgcataccttttcttgccataccatttagccgatcaattgtgctcggcaacagcatgcgatttctta
aatgtcatcgtgatttcttaaatgtcatcgtgatttcttaaatgtcatcgtgatttcttaaatgtcatcgtctcgaggagc
gccggagtataaatagaggcgcttcgtctacggagcgacaattcaattcaaacaagcaaagtgaacacgtcg
ctaagcgaaagctaagcaaataaacaagcgcagctgaacaagctaaacaatctgcagccaagctgatcctct
agggtacgcgctagagtcgagaggcctgtttaaacgatccaccggtcgccaccatggcctcctccgaggacgt
catcaaggagttcatgcgcttcaaggtgcgcatggagggctccgtgaacggccacgagttcgagatcgagggc
gagggcgagggccgcccctacgagggcacccagaccgccaagctgaaggtgaccaagggcggcccccctg
cccttcgcctgggacatcctgtcccccagttccagtacggctccaaggtgtacgtgaagcaccccgccgacat
ccccgactacaagaagctgtccttccccgagggcttcaagtgggagcgcgtgatgaacttcgaggacggcgg
cgtggtgaccgtgacccaggactcctccctgcaggacggctgcttcatctacaaggtgaagttcatcggcgtga
acttcccctccgacggccccgtaatgcagaagaagactatgggctgggagccctccaccgagcgcctgtacc
cccgcgacggcgtgctgaagggcgagatccacaaggccctgaagctgaaggacggcggccactacctggt
ggagttcaagtccatctacatggccaagaagcccgtgcagctgcccggctactactacgtggactccaagctg
gacatcacctcccacaacgaggactacaccatcgtggagcagtacgagcgcgccgagggccgccaccacct
gttcctgtagcggccgcgactctagatcataatcagccataccacatttgtagaggttttacttgctttaaaaaacct
cccacacctccccctgaacctgaaacataaaatgaatgcaattgttgttgttaacttgtttattgcagcttataatggt
tacaaataaagcaatagcatcacaaatttcacaaataaagcatttttttcactgcattctagttgtggtttgtccaaa
ctcatcaatgtatcttaaggcgtaaattgtaagcgttaatgactagtcacgtaataagtgtgcgttgaatttattcgca
aaaacattgcatattttcggcaaagtaaaattttgttgcataccttatcaaaaaataagtgctgcatactttttagaga
aaccaaataatttttattgcatacccgtttttaataaaatacattgcataccctcttttaataaaaaaatattgcatacttt
gacgaaacaaattttcgttgcatacccaataaaagattattatattgcatacccgtttttaataaaatacattgcata
ccctcttttaataaaaaaatattgcatacgttgacgaaacaaattttcgttgcatacccaataaaagattattatattgc
ataccttttcttgccataccatttagccgatcaattctagtatgtatgtaagttaataaaacccttttttggagaatgtag
atttaaaaaaacatattttttttttatttttactgcactggacatcattgaacttatctgatcagttttaaatttacttcgatc
caagggtatttgaagtaccaggttctttcgattacctctcactcaaaatgacattccactcaaagtcagcgctgtttg
cctccttctctgtccacagaaatatcgccgtctctttcgccgctgcgtccgctatctctttcgccaccgtttgtagcgtt
acctagcgtcaatgtccgccttcagttgcactttgtcagcggtttcgtgacgaagctccaagcggtttacgccatca
attaaacacaaagtgctgtgccaaaactcctctcgcttcttatttttgtttgtttttgagtgattggggtggtgattggttt
tgggtgggtaagcaggggaaagtgtgaaaaatcccggcaatgggccaagaggatcaggagctattaattcgc
ggaggcagcaaacacccatctgccgagcatctgaacaatgtgagtagtacatgtgcatacatcttaagttcactt
gatctataggaactgcgattgcaacatcaaattgtctgcggcgtgagaactgcgacccacaaaaatcccaaac
cgcaattgcacaaacaaatagtgacacgaaacagattattctggtagctgttctcgctatataagacaattttttga
gatcatatcatgatcaagacatctaaaggcattcattttcgactatattctttttttacaaaaaatataacaaccagat
attttaagctgatcctagatgcacaaaaaataaataaaagtataaacctacttcgtaggatacttcggggtactttttt
gttcggggttagatgagcataacgcttgtagttgatatttgagatcccctatcattgcagggtgacagcggagcgg
cttcgcagagctgcattaaccagggcttcgggcaggccaaaaactacggcacgctccggccacccagtccgc
cggaggactccggttcagggagcggccaactagccgagaacctcacctatgcctggcacaatatggacatctt
tggggcggtcaatcagccgggctccggatggcggcagctggtcaaccggacacgcggactattctgcaacga
gcgacacataccggcgcccaggaaacatttgctcaagaacggtgagtttctattcgcagtcggctgatctgtgtg
aaatcttaataaagggtccaattaccaatttgaaactcagtttgcggcgtggcctatccgggcgaacttttggccgt
gatgggcagttccggtgccggaaagacgaccctgctgaatgcccttgcctttcgatcgccgcagggcatccaa
gtatcgccatccgggatgcgactgctcaatggccaacctgtggacgccaaggagatgcaggccaggtgcgcc
tatgtccagcaggatgacctctttatcggctccctaacggccagggaacacctgattttccaagccatggtgcgg
atgccacgacatctgacctatcggcagcgagtggcccgcgtggatcaggtgatccaggagctttcgctcagca
aatgtcagcacacgatcatcggtgtgcccggcagggtgaaaggtctgtccggcggagaaaggaagcgtctgg
cattcgcctccgaggctctaaccgatccgccgcttctgatctgcgatgagcccacctccggactggactcctttac
cgcccacagcgtcgtccaggtgctgaagaagctgtcgcagaagggcaagaccgtcatcctgaccattcatca
gccgtcttccgagctgtttgagctctttgacaagatccttctgatggccgagggcagggtagctttcttgggcactcc
cagcgaagccgtcgacttcttttcctagtgagttcgatgtgtttattaagggtatctagtattacataacatctcaactc
ctatccagcgtgggtgcccagtgtcctaccaactacaatccggcggacttttacgtacaggtgttggccgttgtgc
ccggacgggagatcgagtcccgtgatcggatcgccaagatatgcgacaattttgccattagcaaagtagcccg
ggatatggagcagttgttggccaccaaaaatctggagaagccactggagcagccggagaatgggtacaccta
caaggccacctggttcatgcagttccgggcggtcctgtggcgatcctggctgtcggtgctcaaggaaccactcct

cgtaaaagtgcgacttattcagacaacggtgagtggttccagtggaaacaaatgatataacgcttacaattcttg
gaaacaaattcgctagattttagatagaattgcctgattccacacccttcttagttttttcaatgagatgtatagtttat
agttttgcagaagataaataaatttcatttaactcgcgaatattaatgagatgcgagtaacattttaatttgcagatg
gttgccatcttgattggcctcatctttttgggccaacaactcacgcaagtgggtgtgatgaatatcaacggagccat
cttcctcttcctgaccaacatgacctttcaaaacgtctttgccacgataaatgtaagtcatgtttagaatacatttgca
tttcaataatttactaactttctaatgaatcgattcgatttaggtgttcacctcagagctgccagtttttatgagggagg
cccgaagtcgactttatcgctgtgacacatactttctgggcaaaacgattgccgaattgccgctttttctcacagtgc
cactggtcttcacggcgattgcctatccgatgatcggactgcgggccggagtgctgcacttcttcaactgcctggc
gctggtcactctggtggccaatgtgtcaacgtccttcggatatctaatatcctgcgccagctcctcgacctcgatgg
cgctgtctgtgggtccgccggttatcataccattcctgctctttggcggcttcttcttgaactcgggctcggtgccagt
atacctcaaatggttgtcgtacctctcatggttccgttacgccaacgagggtctgctgattaaccaatgggcggac
gtggagccgggcgaaattagctgcacatcgtcgaacaccacgtgccccagttcgggcaaggtcatcctggag
acgcttaacttctccgccgccgatctgccgctggactacgtgggtctggccattctcatcgtgagcttccgggtgct
cgcatatctggctctaagacttcgggcccgacgcaaggagtagccgacatatatccgaaataactgcttgttttttt
ttttaccattattaccatcgtgtttactgtttattgccccctcaaaaagctaatgtaattatatttgtgccaataaaaaca
agatatgacctatagaatacaagtatttccccttcgaacatccccacaagtagactttggatttgtcttctaaccaa
aagacttacacacctgcataccttacatcaaaaactcgtttatcgctacataaaacaccgggatatatttttatata
catacttttcaaatcgcgcgccctcttcataattcacctccaccacaccacgtttcgtagttgctctttcgctgtctccc
acccgctctccgcaacacattcaccttttgttcgacgaccttggagcgactgtcgttagttccgcgcgattcggttcg
ctcaaatggttccgagtggttcatttcgtctcaatagaaattagtaataaatatttgtatgtacaatttatttgctccaat
atatttgtatatatttccctcacagctatatttattctaatttaatattatgactttttaaggtaatttttttgtgacctgttcgga
gtgattagcgttacaatttgaactgaaagtgacatccagtgtttgttccttgtgtagatgcatctcaaaaaaatggtg
ggcataatagtgttgtttatatatatcaaaaataacaactataataataagaatacatttaatttagaaaatgcttgg
atttcactggaactagaattaattcgggcggccgccaccgcggtggagctccaattcgccctatagtgagtcgtat
tacgcgcgctcactggccgtcgttttacaacgtcgtgactgggaaaaccctggcgttacccaacttaatcgccttg
cagcacatccccctttcgccagctggcgtaatagcgaagaggcccgcaccgatcgcccttcccaacagttgcg
cagcctgaatggcgaatgggacgcgccctgtagcggcgcattaagcgcggcgggtgtggtggttacgcgcag
cgtgaccgctacacttgccagcgccctagcgcccgctcctttcgctttcttcccttcctttctcgccacgttcgccgg
ctttccccgtcaagctctaaatcgggggctccctttagggttccgatttagtgctttacggcacctcgaccccaaaa
aacttgattagggtgatggttcacgtagtgggccatcgccctgatagacggtttttcgccctttgacgttggagtcca
cgttctttaatagtggactcttgttccaaactggaacaacactcaaccctatctcggtctattcttttgatttataaggg
attttgccgatttcggcctattggttaaaaaatgagctgatttaacaaaaatttaacgcgaattttaacaaaatattaa
cgcttacaatttag
