# Supplementary material for: A Versatile ΦC31 Based Reporter System for Measuring AP-1 and Nrf2 Signaling in Drosophila and in Tissue Culture
Source: PLoS One. 2012 Apr 11;7(4):e34063. doi: 10.1371/journal.pone.0034063 (PMC3324472; doi:10.1371/journal.pone.0034063)
Supplement: Table S1 — Primers used to generate amplicons for dsRNA synthesis. (DOC) [file pone.0034063.s003.doc]

**Supplemental Table S**1: Primers used to generate amplicons for dsRNA synthesis.

| **Primer name** | **Reagent ID** | **Primer sequence** |
| --- | --- | --- |
| CncCi Forward | HFA15600 Forward | TCAATGTGCTACCTTATTGACT |
| CncCi Reverse | HFA15600 Reverse | TTATCTTGTTGAAGCTCCTCC |
| MafSi Forward | BKN22110 Forward | GATTTGGTGAGCATTTCGGT |
| MafSi Reverse | BKN22110 Reverse | AAACAGGAATGTGACTGGGC |
| Hepi Forward | HFA20337 Forward | GTGGTCCCGGTGGTGGA |
| Hepi Reverse | HFA20337 Reverse | ACCTTGCCCAGGATCTGTT |
| Bski Forward | BKN28156 Forward | AGCATCCACTTCCTCAGCAT |
| Bski Reverse | BKN28156 Reverse | GGTACACCATCACCCTCGTT |
| GFPi Forward | NA | CACATGAAGCAGCACGACTT |
| GFPi Reverse | NA | TGTTCTGCTGGTAGTGGTCG |
| fluci Forward | NA | GGAGAGCAACTGCATAAGGC |
| fluci Reverse | NA | ACATCGACTGAAATCCCTGG |

Each primer sequence is preceded by a T7 promoter (sequence TAATACGACTCACTATAGG) at the 5’ end. The ‘Reagent IDs’ refer to the pre-designed dsRNAs obtained through ‘GenomeRNAi’ database (<http://genomernai.de/GenomeRNAi/f>). Primers for dsRNAs against GFP and Firefly Luciferase (fluc) were designed using E-RNAi webservice.
